# From urban neighbourhood environments to cognitive health: a cross-sectional analysis of the role of physical activity and sedentary behaviours

**DOI:** 10.1186/s12889-021-12375-3

**Published:** 2021-12-23

**Authors:** Ester Cerin, Anthony Barnett, Jonathan E. Shaw, Erika Martino, Luke D. Knibbs, Rachel Tham, Amanda J. Wheeler, Kaarin J. Anstey

**Affiliations:** 1 Mary MacKillop Institute for Health Research, Australian Catholic University, Level 5, 215 Spring Street, Melbourne, Victoria 3000 Australia; 2 School of Public Health, The University of Hong Kong, 7 Sassoon Rd., Sandy Bay, Hong Kong, Hong Kong, SAR China; 3 Baker Heart and Diabetes Institute, Melbourne, VIC Australia; 4 Department of Community Medicine, UiT The Artic University of Norway, Tromsø, Norway; 5 School of Public Health and Preventive Medicine, Monash University, Melbourne, VIC Australia; 6 School of Life Sciences, La Trobe University, Melbourne, VIC Australia; 7 School of Population and Global Health, University of Melbourne, Melbourne, VIC Australia; 8 School of Public Health, The University of Sydney, Camperdown, NSW Australia; 9 Centre for Air Pollution, Energy and Health Research, Glebe, NSW Australia; 10 Menzies Institute for Medical Research, College of Health and Medicine, University of Tasmania, Hobart, TAS Australia; 11 School of Psychology, University of New South Wales, Randwick, NSW Australia; 12 Neuroscience Research Australia (NeuRA), Sydney, Australia

**Keywords:** walkability, greenspace, blue space, physical activity, sedentary behaviours, cognitive function

## Abstract

**Background:**

There is a dearth of studies on the effects of the neighbourhood environment on adults’ cognitive function. We examined how interrelated aspects of the built and natural neighbourhood environment, including air pollution, correlate with adults’ cognitive function, and the roles of physical activity and sedentary behaviours in these associations.

**Methods:**

We used data from 4,141 adult urban dwellers who participated in the Australian Diabetes, Obesity and Lifestyle 3 study on socio-demographic characteristics, neighbourhood self-selection, physical activity and sedentary behaviours, and cognitive function. Neighbourhood environmental characteristics included population density, intersection density, non-commercial land use mix, and percentages of commercial land, parkland and blue space, all within 1 km residential buffers. We also calculated annual mean concentrations of NO_2_ and PM_2.5_. Generalised additive mixed models informed by directed acyclic graphs were used to estimate the total, direct and indirect effects of environmental attributes on cognitive functions and the joint-significance test was used to examine indirect effects via behaviours.

**Results:**

In the total effects models, population density and percentage of parkland were positively associated with cognitive function. A positive association of PM_2.5_ with memory was also observed. All neighbourhood environmental attributes were directly and/or indirectly related to cognitive functions via other environmental attributes and/or physical activity but not sedentary behaviours. Engagement in transportation walking and gardening frequency partially mediated the positive effects of the neighbourhood environment on cognitive function, while frequency of transportation walking mediated the negative effects.

**Conclusions:**

In the context of a low-density country like Australia, denser urban environments with access to parkland may benefit residents’ cognitive health by providing opportunities for participation in a diversity of activities. A more fine-grained characterisation of the neighbourhood environment may be necessary to tease out the negative and positive impacts of inter-related characteristics of urban neighbourhood environments on cognitive function.

**Supplementary Information:**

The online version contains supplementary material available at 10.1186/s12889-021-12375-3.

## Background

Increases in the number and proportion of older adults are widespread demographic trends with major implications for nearly all sectors of society [1]. According to the United Nations, the global number of people aged 60 years and older has more than doubled in the last 35 years and is projected to follow this trend in the next three decades, reaching 2.1 billion in 2050 [1]. As populations grow older, governments need to devise evidence-based, large-scale strategies and policies focused on the promotion of older people’s well-being and productivity through the maintenance of their health and cognitive function.

The physical and social characteristics of urban residential neighbourhoods have been identified as major determinants of morbidity and mortality [2]. They are especially important to ageing populations whose mobility is often limited, making them more reliant on their immediate environment for daily living. Neighbourhood environment design has been linked to health-related lifestyle behaviours, environmental exposures and physical health conditions known to affect the risk of cognitive decline and dementia, including physical inactivity and air pollution exposure [3]. For example, higher levels of residential density, street connectivity and access to parks and a variety of services may promote utilitarian walking [4] and engagement in leisure-time physical activity [5] in older populations and, by doing so, displace some of the time spent being sedentary [6, 7]. Yet, while the impacts of neighbourhood characteristics on middle-age and older adults’ active lifestyle have been relatively extensively researched, the same cannot be said for impacts on cognitive function and the mediating role of physical activity and sedentary behaviours in these associations [8, 9].

A small number of studies have examined the associations of a limited range of characteristics of the neighbourhood social, built and/or natural environment with cognitive function [8, 10]. While a few of these studies found that access to services and public transport were linearly and positively related to better cognitive function and brain health [8, 11, 12], others observed curvilinear or even negative relationships [13, 14]. Contradictory or mixed findings have also been reported with regards to the effects of the natural environment (e.g., access to green areas) [10, 14] and air pollution [15–17], although the latter category of exposures tended to be more consistently negatively related to cognitive health. These discrepant findings may be in part due to unadjusted environmental confounders, which is a common issue in the published literature [18]. For example, the failure to include traffic-related air pollution, often higher in high-density neighbourhoods [16, 17], in studies of neighbourhood environment and cognitive health may result in the underestimation of positive effects of destination accessibility on cognitive function. Similarly, failing to account for destination accessibility may attenuate the estimated negative effects of air pollution on cognitive function [9]. A more robust assessment of the potential effects of the neighbourhood environment on cognitive health relies on the inclusion of multiple key environmental exposures and a careful consideration of their inter-relationships in models of cognitive function, as proposed in a recent ecological model of the effects of urban environments on cognitive health [9, 18] and in line with earlier recommendations [19].

**Fig. 1:**
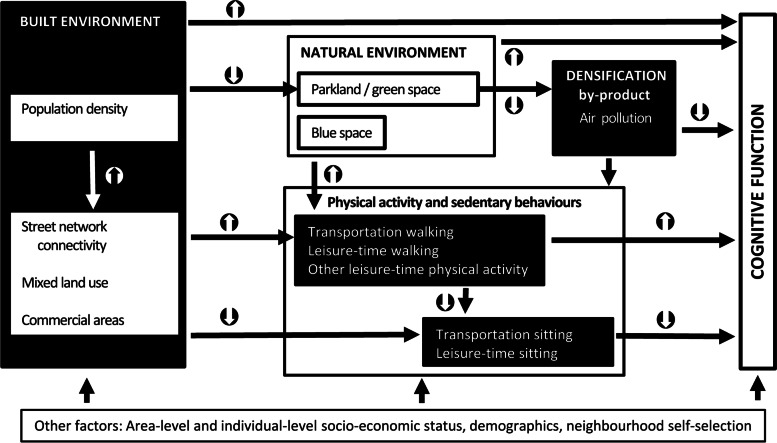
A simplified ecological model of neighbourhood environmental influences on cognitive function. ↑ indicate positive associations; ↓ indicate negative associations

According to this model, interrelated characteristics of the urban environment are deemed to influence cognitive health indirectly by shaping travel (e.g., engagement in active vs. motorised transport) and other lifestyle behaviours (e.g., leisure-time physical activity or gardening) and, more directly, by exposing residents to potentially harmful factors (e.g., air pollution) and/or limiting their exposures to natural environments (e.g., greenness or blue spaces) that promote restauration and social contacts [9, 18, 20]. Adopting this ecological framework, the present study examined how interrelated aspects of the built and natural neighbourhood environment, including air pollution as a sequela of urban densification, correlate with cognitive function in mid-age and older Australians, and the extent to which these relationships are explained by physical activity and sedentary behaviours that have been previously linked [3], or are potentially linked [21, 22], to cognitive health (Fig. 1). To achieve this, we examined the total cross-sectional effects of environmental attributes on cognitive function as well as the indirect (behaviour-mediated) and direct (unmediated) effects. In general, we hypothesised that neighbourhood built environment indicators of densification (i.e., population density, street intersection density and access to various destinations) would not only show potentially beneficial effects on cognitive function via transportation [3, 4, 23] and leisure-time activities [3, 5, 23], but also detrimental effects via poorer access to parks [7, 10], higher levels of air pollutants [9, 11, 18] and their impacts on leisure-time activities [5, 24, 25] (Fig. 1). In testing these hypotheses and unlike previous research [9], this study modelled interrelationships between neighbourhood environmental attributes (e.g., accounted for the fact that increases in population density typically lead to increases in commercial destinations, street connectivity and air pollution [26]) enabling a more robust estimation of, and a distinction between, the total and direct effect of aspects of the environment on cognitive function as well as on physical activity and sedentary behaviours.

## Methods

### Study design and participants

This study used secondary data from the Australian Diabetes, Obesity and Lifestyle study (AusDiab), a population-based longitudinal survey designed to examine the prevalence, incidence and determinants of diabetes in Australians aged 25 years and older [27]. AusDiab was established in 1999 with 11,247 adults participating in the 1999-2000 baseline study. Two-stage stratified sampling was used to recruit participants from 42 areas in the metropolitan and regional cities of six Australian states and the Northern Territory (response rate for AusDiab survey component relevant to this paper: 67%). A study area consisted of contiguous census administrative units. Participants were eligible to participate if they had no physical or intellectual disabilities and resided at their addresses for 6 months or longer prior to the survey. Two follow-up assessments were subsequently conducted, one in 2004–05 (AusDiab2; retention rate = 59.3%) and the other in 2011–12 (AusDiab3; retention rate from baseline = 44.6%). Participants who took part in AusDiab3 were younger (*p*<.001) and more physically active (*p*<.01) at baseline than those who dropped out from the study. Further, they were less likely to smoke (*p*<.01) and had higher levels of educational attainment (*p*<.001). Loss to follow-up due to death was 9.5% and 15% in AusDiab2 and AusDiab3, respectively. Further details on the AusDiab data collection procedures have been provided elsewhere [27, 28]. The AusDiab study was approved by the Alfred Hospital Ethics Committee (no. 39/11). Written informed consent was obtained from all participants.

Environmental exposure and cognitive function data at baseline and for AusDiab2 were unavailable. Hence, the present study utilized data from the participants who took part in AusDiab 3, when measurement of cognitive function was conducted on those who attended testing sites for the biomedical examination (n=4614). As this study focused on the potential effects of urban neighbourhood environments on cognitive function, 473 participants who did not reside in significant urban areas – namely, significant towns and cities of 10,000 people or more [29] - were excluded from the analyses. The analytical sample of the present study consisted of 4141 participants (1855 men and 2286 women) residing in 1,286 Statistical Areas 1 (SA1), the smallest administrative units for the release of census data. Table 1 reports the characteristics of the analytic sample.

**Table 1:** Sample characteristics (*N* = 4,141)

Characteristics	Statistics	Characteristics	Statistics
***Socio-demographic characteristics***			
Age, years, M ± SD	61.1 ± 11.4	Sex, female, %	55.2
Educational attainment, %		Employment status, %	
Up to secondary	32.7	Not employed	30.4
Trade, technician certificate	29.1	Paid employment	52.2
Associate diploma & equiv.	14.5	Volunteering	15.1
Bachelor degree, post-graduate diploma	23.1	Missing data	2.3
Missing data	0.6	Area-level IRSAD, M ± SD	6.4 ± 2.7
Living arrangements, %		English-speaking background, %	89.9
Couple without children	48.2	Household income, annual, %	
Couple with children	26.8	Up to $49,999	32.9
Other	22.4	$50,000 - $99,999	26.8
Missing data	2.4	$100,000 and over	28.9
		Does not know or refusal	8.8
		Missing data	2.7
Residential self-selection – access to destinations*,* M ± SD	3.0 ± 1.4	Residential self-selection – recreational facilities*,* M ± SD	3.1 ± 1.5
Missing data, %	7.8	Missing data, %	7.8
***Physical activity and sedentary behaviour***			
Transportation walking		Leisure-time walking	
Times per week*,* M ± SD	1.4 ± 3.5	Times per week, M ± SD	2.4 ± 2.5
Prevalence, %	29.1	Prevalence, %	61.6
Missing data, %	2.7	Missing data, %	3.0
Vigorous gardening		Resistance training	
Times per week*,* M ± SD	0.8 ± 1.5	Times per week, M ± SD	0.9 ± 2.3
Prevalence, %	37.1	Prevalence, %	25.5
Missing data, %	2.6	Missing data, %	2.6
Sitting for transport, h/day*,* M ± SD	0.8 ± 0.8	Leisure-time sitting, h/day*,* M ± SD	2.6 ± 1.6
Missing data, %	2.7	Missing data, %	2.8
Sitting (other purposes), h/day*,* M ± SD	3.4 ± 2.4		
Missing data, %	2.9		
***Health-related variables***			
Tobacco-smoking status, %		Heart problems/stroke (past), %	8.7
Current smoker	7.0	Missing data, %	1.0
Previous smoker	35.9		
Non-smoker	54.5		
Missing data	2.6		
***Cognitive function*,** M ± SD			
Memory, CVLT score	6.5 ± 2.4	Processing speed, SDMT score	49.7 ± 11.6
Missing data, %	2.3	Missing data, %	2.0
***Neighbourhood environmental characteristics (1km-radius street-network buffers)****,* M ± SD*			
Population density, persons/ha	17.4 ± 10.0	Street intersection density, intersections/km^2^	62.2 ± 32.2
Percentage of commercial land use in residential buffer	2.5 ± 6.1	Non-commercial land use mix, entropy score (0 to 1)	0.14 ± 0.13
Percentage of parkland in residential buffer	11.6 ± 12.5	Percentage of blue space (waterbody) in residential buffer	0.24 ± 1.98
NO_2_, ppb	5.5 ± 2.1	PM_2.5_, μg/m^3^	6.3 ± 1.7

*M *mean; *SD *standard deviation; *IRSAD *Index of Relative Socioeconomic Advantage and Disadvantage; *CVLT* California Verbal Learning Test; *SDMT* Symbol–Digit Modalities test; *NO*_2_ nitrogen dioxide; *PM*_2.5_ particulate matter < 2.5 μm; *neighbourhood environmental characteristics had no missing data

### Measures

#### Environmental exposures

Measures of the neighbourhood built and natural environment were generated using ESRI’s ArcGIS v.10.5 software (ESRI, Redlands). Participants’ residential addresses were geocoded and 1-km untrimmed street-network buffers were created around the geocoded locations following procedures employed in international studies of neighbourhood environmental determinants of health [7, 30, 31]. A 1-km radius was used to create residential buffers because it corresponds to the distance that adults and older adults without mobility problems can cover in a 10-20 minute walk [30], and the latter is commonly used to define a neighbourhood [32, 33]. Four built environment and two natural environment measures were computed for each participant’s residential buffer. These were population density (persons/ha), street intersection density (intersections/km^2^), percentage of commercial land use, an entropy score denoting the heterogeneity of non-commercial land use (range: 0-1), percentage of parkland and percentage of blue spaces (e.g., lakes, coastlines, rivers and reservoirs). Data sources were the Australian Bureau of Statistics (ABS) Mesh Block data from the 2011 Census [34], the PSMA Australia’s 2012 Transport & Topography dataset [35] and the national topographic spatial data for surface water features sourced from Geoscience Australia [36]. Further details on the data sources, measures and justifications for including them in this study are provided in the Additional file 1 (S1).

Two ambient air pollutants, nitrogen dioxide (NO_2_, units: ppb) and fine particulate matter < 2.5 μm in aerodynamic diameter (PM_2.5_, units: μg/m^3^), that have been linked to cognitive ill-health [17, 37, 38] were employed in this study and exposures were estimated at each residential address. Both pollutants were estimated using satellite-based land-use regression models, which use spatial predictors of annual average NO_2_ and PM_2.5_ at fixed-site monitors (e.g., roads, industrial emissions, wood-fired heater smoke), including time-varying information from satellites, to predict concentrations at unmeasured locations (e.g., residential addresses). The models were used to predict exposure at the time of the AusDiab 3 study. The development and validation of the models is described in detail elsewhere [39–41]; the NO_2_ model captured 81% of spatial variability in annual NO_2_ (RMSE: 1.4 ppb), while the PM_2.5_ model captured 63% of spatial variability (RMSE: 1 μg/m^3^).

#### Cognitive function (outcomes)

Two cognitive function outcomes were examined: memory and processing speed. Memory is the ability to encode, store, retain and recall information. It is essential to learning and, as such, is one of the most important cognitive functions. Although memory typically declines with age, its decline can be slowed down by regularly engaging in physical, social and intellectual activities [23]. Processing speed is a cognitive ability defined as the time needed to perform a mental task, which also declines with age [23]. It represents one of most important skills in learning, academic performance, and reasoning. Slow processing speed may have negative effects on executive functions, such as planning, decision-making, goal-setting and attention. Information processing speed may be maintained by engaging in intellectually stimulating activities, physical activity and reducing exposure to cardiovascular risk factors [23].

In this study, the California Verbal Learning Test (CVLT) was used to assess *memory* [42]. The number of 16 common shopping list items correctly recalled after a 20-minute delay following their presentation represented the score on the test. The Symbol–Digit Modalities Test (SDMT) was employed to assess *processing speed* [43]. Participants used a reference key to find and orally report the numbers (from 1 to 9) corresponding to nine geometric figures as quickly as possible. The score ranged from 0 to 60 and represented the number of correct responses given in 90 seconds. Both tests have been extensively validated.

#### Physical activity and sedentary behaviours (mediators)

Potential mediators of environment-cognitive function associations included four measures of physical activity adapted from the Active Australia survey [44]: previous-week frequencies of engagement in transportation walking, leisure-time walking, vigorous gardening and resistance training. Two measures of sedentary behaviour based on items developed for AusDiab3 were also included: previous-week average daily hours of sitting time for transport and leisure. This study distinguished between various domains/types of physical activity and sedentary behaviours rather than using measures of total physical activity and total sitting time as potential mediators of environment-cognitive function associations because different environmental characteristics are likely to impact on different domains/types of physical activity and sitting time [4, 5]. Also, there is a dearth of information on the potential effects of different types of physical activity and sedentary behaviours on cognitive functions. A more detailed description of the measures used in this study is given in the Additional file 1 (S2).

#### Confounders and covariates

A range of additional variables were considered as potential confounders or covariates (here, covariate is defined as a variable that does not necessarily confound an association but explains residual outcome variance) as appropriate (see Additional file 1, Table S1). These included self-reported sex, age, educational attainment, employment status (not working; paid employment; volunteer), household income, living arrangements (living with partner and no children; living with partner and children; living alone; other living arrangements), ethnicity (English-speaking background vs. non-English speaking background), history of heart problems or stroke, tobacco smoking status (current smoked; past smoker; never smoker), and average daily hours of sitting for occupational purposes and purposes other than those listed as potential mediators (see previous section). To account for the confounding effects of residential self-selection – namely, people selecting to live in areas providing the facilities that satisfy their preferred lifestyle [45, 46] – two variables based on participants’ responses to 5-point-scale items assessing the importance of reasons for choosing to live in the current neighbourhood [46, 47] were included: one related to access to recreational facilities (1 item), the other related to good access to various destinations (4 items). Area-level socio-economic status quantified using the 2011 Index of Relative Socioeconomic Advantage and Disadvantage (IRSAD) [48] was also considered a potential area-level confounder or covariate, as appropriate.

#### Analytical Plan and Hypotheses

Descriptive statistics and percentage of missing values were computed for all variables. Over 17% of cases had missing data on at least one variable and 4.5% on more than three variables. As data were not missing completely at random (see Additional file 1; S3), ten imputed datasets were created for the regression analyses. Multiple imputations by chained equations were performed using the package ‘mice’ [49] in R version 4.0.0 [50]. Generalised additive mixed models (GAMMs; package ‘mgcv’ version 1.8.22 [51] in R) with random intercepts at the SA1 level were used to estimate cross-sectional total, direct and indirect effects of environmental attributes on cognitive function [51]. Here, the meaning of ‘effect’ (common in mediation analyses) needs to be interpreted in the context of the cross-sectional observational nature of the study with possible unmeasured confounders. Directed acyclic graphs (DAGs) informed the selection of a minimal sufficient set of confounders to be included in the GAMMs estimating exposure-outcomes, exposure-mediators and mediators-outcomes relationships (Figure S1). The DAGs were based on the hypothesised causal effects among the variables according to previous studies and the authors’ expert opinion. Potential multicollinearity was assessed by computing the Variance Inflation Factor (VIF) for each variable included in the GAMMs. All VIFs were smaller than 3, indicating no collinearity issues [52]. Analyses were conducted in several steps which are described in detail in the Additional file 1 (S3 and Table S1).

#### Total effects of neighbourhood environmental characteristics on cognitive function

The total effects of neighbourhood characteristics on each of the two cognitive function outcomes (memory and processing speed) were first estimated as detailed in the Additional file 1 (Steps 1Ta to 1Th in Table S1). We hypothesised that neighbourhood population density, street intersection density, percentage of commercial land use and non-commercial land use mix would be positively related to both measures of cognitive function, as they provide opportunities for physical, social and cognitive activities to residents [8, 9, 18]. This hypothesis was based on air pollution levels being typically low in Australia [53] and the assumption that increases in air pollution associated with densification would not be sufficiently high to offset the positive effects of environmental complexity and access to destinations. The total effects of percentage of parkland and blue space in the neighbourhood on cognitive function were expected to be positive or, as sometimes observed, curvilinear [8, 9, 18], and those of air pollution negative [16, 17].

#### Mediated and direct effects of neighbourhood environmental characteristics on cognitive function

Mediation was examined using the joint-significance test [26, 54] according to which data support mediation if the associations between an exposure and its mediator(s) and the exposure-adjusted associations between the mediator(s) and the outcome are both statistically significant (*p*<.05). Figure 1 depicts most of the hypothesised direct and indirect effects between environmental exposures, physical activity and sedentary behaviours, and cognitive function based on the current literature (see Background). A detailed description of the models used to estimate mediated and direct effects and related hypotheses can be found in the Additional file 1 (S3 and Table S1).

## Results

Table 1 shows the socio-demographic characteristics of the analytic sample. The mean (±SD) age was 61 ± 11 years (range: 34 – 97 years). Approximately 52% of participants were employed and 90% were of English-speaking background. Nearly a quarter had tertiary education and lived with a partner/spouse and children. The sample was heterogeneous in socio-economic status, with 32% and 29% of participants reporting respectively an annual household income below $50,000 and above $100,000. Leisure-time walking was the most prevalent type of physical activity (62%), followed by vigorous gardening (37%). On average, participants spent most of their sitting time for work and purposes other than transportation and leisure (3.4 ± 2.4 h/day). They spent an average of 2.6 h/day sitting for leisure, including TV watching and computer time, and 0.8 h/day sitting for transportation. The relative variability of environmental attributes was substantial. For example, population density ranged from 0.5 to 146 persons/ha and non-commercial land use mix from 0 to 0.66 (theoretical range 0 to 1). The average percentage of residential-buffer land devoted primarily to commercial use (2.5%) was lower than parkland (11.6%). The annual average concentrations of air pollutants were low, with NO_2_ and PM_2.5_ reaching 5.5 ppb and 6.3 μg/m^3^, respectively.

### Total and Direct Effects of Environmental Exposures on Cognitive Function

Estimates of the total and direct effects of environmental exposures on cognitive function are summarised in Table 2. In the total-effect models, higher levels of population density and annual concentrations of PM_2.5_ were associated with higher scores on the memory test. However, only the latter effect remained significant after adjustment for potential mediators, albeit attenuated (see direct effect estimates in Table 2). Percentage of parkland in residential buffers was positively associated with scores on the memory as well as processing speed test in both total- and direct-effect models. While no other statistically significant total effects were observed, a significant direct effect of annual average concentrations of NO_2_ on processing speed was found.

**Table 2:** Total and direct effects of neighbourhood environmental characteristics on cognitive function

Environmental characteristic (units)	Effect	Memory (CVLT raw score)	Processing speed (SDMT raw score)
		***b*** (95% CI)	***b*** (95% CI)
Population density (10 persons/ha)	Total	**0.121 (0.045, 0.197)**	0.151 (-0.146, 0.447)
	Direct	0.069 (-0.045, 0.184)	-0.064 (-0.505, 0.378)
Street intersection density (10 intersections/km^2^)	Total	-0.011 (-0.288, 0.286)	-0.026 (-0.137, 0.086)
	Direct	0.007 (-0.022, 0.037)	-0.001 (-0.114, 0.113)
Percentage of commercial land use (10%)	Total	-0.004 (-0.124, 0.116)	0.109 (-0.365, 0.583)
	Direct	-0.054 (-0.176, 0.069)	-0.073 (-0.553, 0.407)
Non-commercial land use mix (0.10 score)	Total	-0.002 (-0.064, -0.061)	0.118 (-0.114, 0.349)
	Direct	-0.014 (-0.078, 0.050)	0.111 (-0.121, 0.342)
Percentage of parkland (10%)	Total	**0.078 (0.014, 0.142)**	**0.243 (0.006, 0.480)**
	Direct	**0.078 (0.014, 0.141)**	**0.211 (0.021, 0.401)**
Percentage of blue space (10%)	Total	-0.150 (-0.490, 0.189)	0.225 (-1.154, 1.604)
	Direct	-0.097 (-0.436, 0.243)	0.492 (-0.886, 1.870)
NO_2_ (ppb)	Total	0.011 (-0.040, 0.061)	0.159 (-0.029, 0.347)
	Direct	0.007 (-0.043, 0.057)	**0.218 (0.032, 0.404)**
PM_2.5_ (μg/m^3^)	Total	**0.096 (0.046, 0.146)**	0.013 (-0.171, 0.197)
	Direct	**0.085 (0.035, 0.135)**	-0.059 (-0.238, 0.121)

*CVLT* California Verbal Learning Test; *SDMT* Symbol-Digit Modalities Test; *b* regression coefficient; *CI* confidence intervals. Total-effect models are not adjusted for physical activity and sedentary behaviour mediators, while direct-effect models are. Statistically significant effects in bold (*p*<.05). Details on regression models, including confounders, are in the Additional file 1 (Table S1)

### Effects of Environmental Exposures on Cognitive Function Mediated by Physical Activity and Sedentary Behaviours (Indirect Effects)

**Fig. 2:**
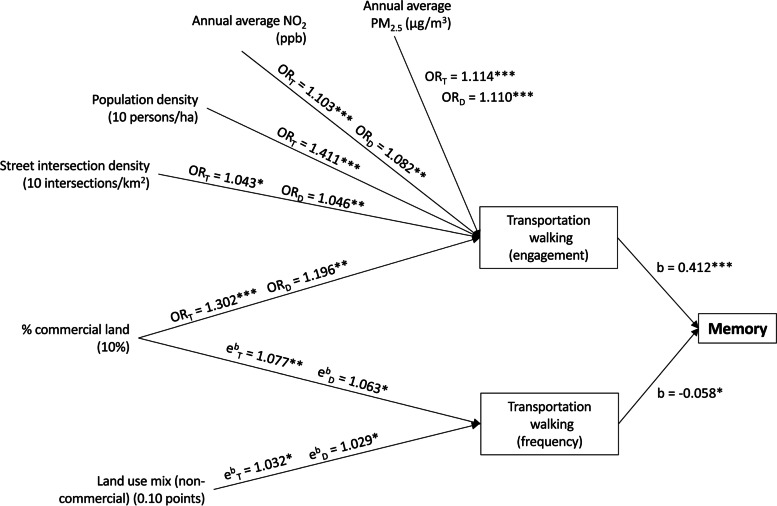
Effects of neighbourhood environmental characteristics on memory mediated by transportation walking. Arrows linking variables indicate significant associations. Transportation walking (frequency) refers to those who engaged in this type of activity. OR, odds ratio; b, regression coefficient; e^b^, exponentiated regression coefficient; subscript D, estimate of direct effect; subscript T, estimate of total effect; ha, hectare; * *p*<.05; ** *p*<.01; *** *p*<.001. All significant and non-significant associations (regression coefficients and 95% CIs) are presented in the Additional file 1 (section S4; Tables S3-S5)

**Fig. 3:**
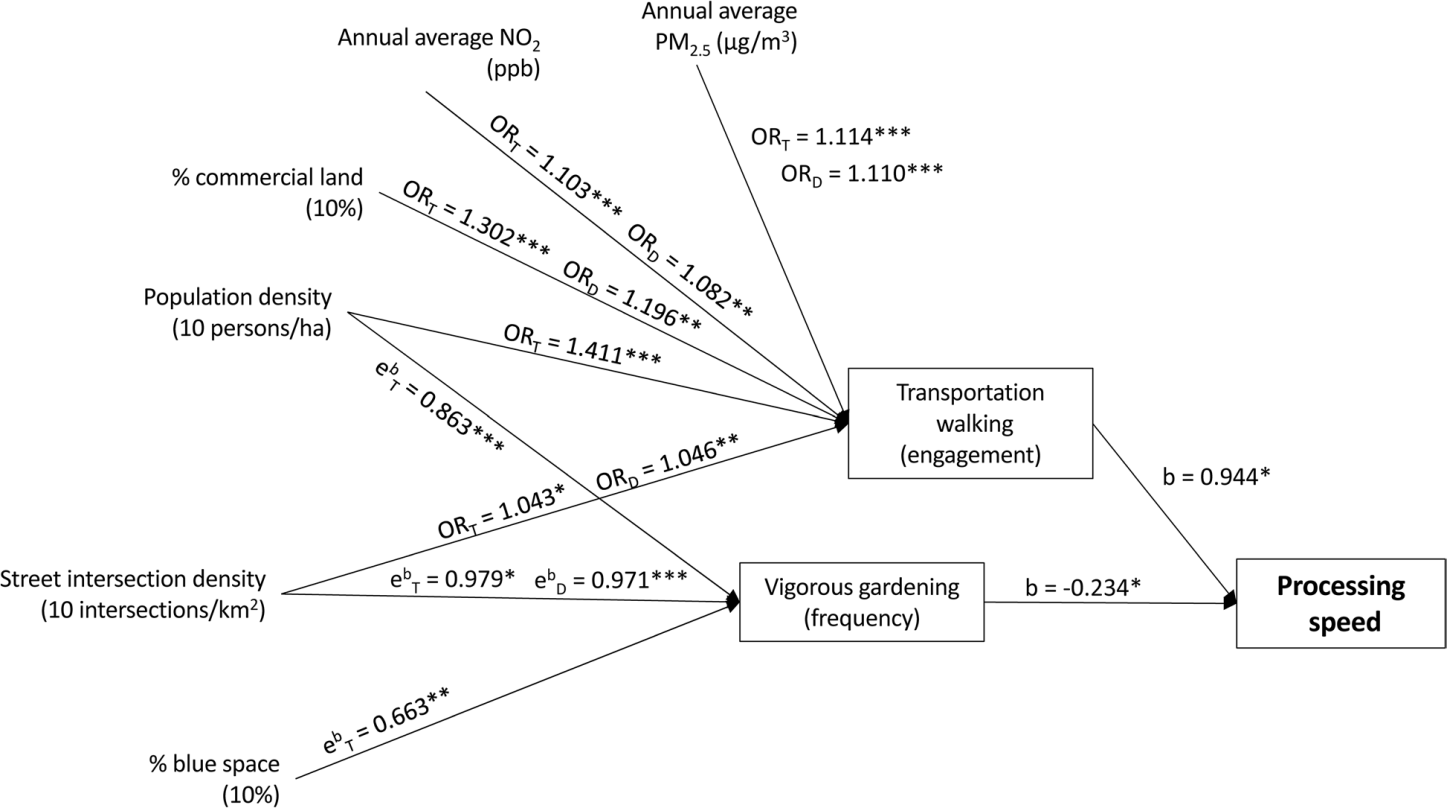
Effects of neighbourhood environmental characteristics on processing speed mediated by transportation walking and vigorous gardening. Arrows linking variables indicate significant associations. Vigorous gardening (frequency) refers to those who engaged in this type of activity. OR, odds ratio; b, regression coefficient; e^b^, exponentiated regression coefficient; subscript D, estimate of direct effect; subscript T, estimate of total effect; ha, hectare; * *p*<.05; ** *p*<.01; *** *p*<.001. All significant and non-significant associations (regression coefficients and 95% CIs) are presented in the Additional file 1 (section S4; Tables S3-S5)

The analysis of indirect effects of environmental characteristics on cognitive function revealed potential pathways of influence through physical activity but not sedentary behaviours (see Figure 2 and 3; Additional file 1, S4; Tables S2-S5). Engagement in transportation walking and frequency of transportation walking in those who engaged in this type of activity were the only potential physical activity mediators of environment-memory associations, with the former measure showing a positive association and the latter a negative association (Figure 2 and Table S5). We estimated that the positive effects of engagement in transportation walking on memory were positive and significant only in those reporting 1 to 4 days/week of transportation walking and not significant in those reporting 5 or more days/week of the same activity.

Higher levels of percentage of commercial land use, street intersection density, NO_2_ and PM_2.5_ independently contributed to higher scores on the memory test via engagement in transportation walking, which was positively related to memory and the aforementioned environmental attributes (see direct effects on transportation walking in Figure 2, estimates with subscript D). Percentage of commercial land use and non-commercial land use mix displayed negative indirect effects on memory through frequency of transportation walking in those who walked for transport (Figure 2). As noted above, in practice, this meant that the higher frequency of transportation walking due to higher levels of commercial land and land use mix resulted in smaller memory gains from engagement in this type of physical activity.

Although, population density and percentage of parkland did not show significant direct effects on transportation walking measures, they had a potential indirect impact on memory through their effects on other environmental attributes (see Table S2, Figure S2) that were directly related to transportation walking (Figure 2). This is why, for example, the total effects (estimates with subscript T in Figure 2) of population density on transportation walking were positive despite the corresponding direct effect not being significant. Specifically, all the effects of population density on memory via transportation walking were mediated by other environmental attributes (street intersection density, percentage of commercial land, non-commercial land use mix and air pollution) (Figure S2) that were directly related to transportation walking (Figure 2). Percentage of blue space was the only environmental attribute not directly or indirectly related to memory (Figures 2 and S2).

Two physical activity measures mediated the associations between neighbourhood environmental characteristics and processing speed (Figure 3). These were engagement in transportation walking and frequency of vigorous gardening in those who engaged in gardening, with the latter measure showing a negative rather than positive association with processing speed. Frequency of resistance training among those engaging in this activity was also positively associated with processing speed (Table S5) but was not a mediator of environment-processing speed associations (Tables S3 and S4).

Environmental characteristics showed positive effects on processing speed via engagement in transportation walking in a similar fashion to that observed for memory (see section above on memory) (Figure 3). In addition, street intersection density and percentage of blue space displayed positive indirect effects on processing speed through frequency of vigorous gardening among those who undertook this activity. In fact, these environmental attributes were negatively related to frequency of gardening which, in turn, was negatively related to processing speed.

Other physical activity (leisure-time walking, resistance training, engagement in vigorous gardening) and sedentary behaviours (sitting for leisure and sitting for transportation) were associated with several environmental characteristics but were not related to cognitive function (Table S4; Figure S6) and, hence, were not potential mediators of environment-cognitive function associations. Findings on environmental correlates of these physical activity and sedentary behaviour measures are reported in the Additional file 1 (section S4; Tables S3 and S4; Figure S6).

## Discussion

Unlike previous research on the neighbourhood environment and cognitive function [8, 55], this study examined the potential effects of characteristics of the neighbourhood built and natural environment in conjunction with ambient air pollution. In doing so, it accounted for the inter-relationships between environmental factors and examined the mediating role of physical activity and sedentary behaviours. We discuss the findings starting from the environmental attributes more proximal to cognitive function (ambient air pollution) as depicted in the proposed conceptual model of neighbourhood environmental influences on cognitive health (Figure 1).

### Air Pollution

This study included annual average PM_2.5_ and NO_2_ estimates in the exposures because higher levels of these air pollutants have been associated with a higher risk of dementia [3] and lower levels of, and faster decline in, cognitive performance [17, 56]. Contrary to expectations, we found positive total and direct effects of PM_2.5_ on memory, and positive direct effects of NO_2_ on processing speed. Positive indirect effects of both pollutants on both cognitive functions mediated by engagement in transportation walking were also observed in the current study.

The unexpected positive effects of PM_2.5_ and NO_2_ on cognitive function could be partly attributed to low levels of air pollution in conjunction with using a coarse measure of access to destinations supporting cognition-enhancing activities (e.g., places for physical activity and socialising). The average values of PM_2.5_ and NO_2_ observed in this study were less than half those found in the U.S. and Europe [57], with the maximum value of annual average PM_2.5_ falling within the lowest quantiles reported elsewhere [58, 59]. The annual average levels of PM_2.5_ and NO_2_ in urban Australia may be too low to show a marked systematic detrimental effect on cognitive function cancelling out the cognitive benefits of living in dense environments with plentiful opportunities for physical and other activities [9]. Ambient concentrations of PM_2.5_ and NO_2_ in urban environments are largely driven by densification, traffic and human activity, including hospitality and entertainment. Although, in this study, the effects of air pollutants were adjusted for population density and indicators of destination accessibility, the indicators used (non-commercial land use mix and percentage of commercial land) were likely unable to differentiate between destinations that promote cognition-enhancing activities from those that do not. Commercial areas that facilitate engagement in cognition-enhancing activities (social, cultural and physical activities) are likely associated with higher levels of pollution (from food preparation; high volumes of visitors and, hence, traffic) than those that do not [39–41]. Such residual confounding may be responsible for the observed positive effects of PM_2.5_ on memory, of NO_2_ on procession speed, and for the positive indirect effects of both air pollutants on cognitive functions mediated by transportation walking. In fact, transportation walking relies on the presence of destinations of daily living and opportunities for various activities in the neighbourhood [4]. Hence, the positive association between neighbourhood ambient air pollution and transportation walking in the present study suggests that the presence of relevant destinations in a neighbourhood was not adequately captured by land use measures.

### Natural Environment

Whilst this study did not find evidence of total and direct effects of neighbourhood blue space on cognitive functions, significant positive total effects of percentage of parkland on both cognitive functions were observed. Parkland accessibility was also directly positively related to memory after adjustment for other environmental attributes, physical activity and sedentary behaviours. Systematic reviews on green space on cognitive function have reported beneficial effects in adults [10], whereas the findings on older adults were inconsistent, especially those from cross-sectional studies [60]. These inconsistences have been in part attributed to self-selection bias and residual SES confounding [10, 62], which we more robustly addressed than previous cross-sectional studies.

The present study suggests positive effects of exposure to greenspace on cognition mainly via mechanisms other than physical activity and sedentary behaviours, such as attention restoration [60] or socialising [20]. For example, according to the attention restoration theory, exposure to nature facilitates the restoration of directed attention depleted by attentional tasks required in urban daily life [60]. That this study did not find leisure-time physical activity, such as recreational walking and resistance training, to be mediators of parkland-cognition relationships may be due to several reasons. First, we did not measure park quality, which is known to impact on park visitation [5]. A positive association between percentage of parkland and engagement in resistance training was observed but the latter was not related to cognitive function. The beneficial effects of resistance training on cognitive function may depend on the volume and intensity of the activity as well as its social context (excising alone or with others) [61]. In fact, we found that frequency of resistance training in those participating in this activity was positively related to processing speed. However, percentage of parkland was unrelated to frequency of resistance training. That leisure-time walking was not positively related to percentage of parkland is somewhat in line with previous studies on environmental correlates of older adults’ leisure-time physical activity [5].

Evidence from the limited number of studies that examined the potential impact of blue spaces on physical activity suggests that they promote an active lifestyle [62]. However, we did not find positive direct effects of blue spaces on walking and resistance training. This could be due to the way blue spaces were operationalised. Several studies used objective distance or perceived access to water bodies as measures of blue space accessibility [63], while we used percentage of blue space area within a 1-km radius residential buffer. Yet, the negative effect of blue space on leisure-time sitting observed in this study suggests that individuals living close to water bodies may engage in other physical activities (e.g., water sports, ball games) that help them to reduce the time they spend on sedentary leisure pursuits [7].

The present study found a negative association between percentage of blue space and frequency of vigorous gardening in those engaging in such activity, which, in turn, was negatively related to processing speed. Residential areas with a larger percentage of blue space are likely to have fewer and/or smaller gardens, and smaller gardens require less maintenance time. Alternatively, residents of areas with access to blue spaces (e.g., beach) may prefer spending more time on a variety of leisure activities rather than frequently engaging in gardening. The negative association between gardening frequency and processing speed suggests that individuals who spend a lot of time gardening may do this at the expense of diversity of activities and, thus, harm their cognitive health [64]. Gardening is usually a solitary or family activity that, if done too frequently, may reduce the opportunities to socialise with a larger circle of people, a cognition-friendly activity [23].

### Built Environment

This study found a positive total effect of population density on memory that were fully mediated by other environmental attributes accompanying densification (all environmental attributes except for blue space) and engagement in transportation walking. As Australia has low levels of air pollution, which is the main health-harming by-product of urbanisation, a positive total effect of population density on cognitive function was expected because dense, complex, destination-rich environments provide opportunities for social contacts, physical activity and other cognition-enhancing activities [9]. In fact, positive associations of population density with street intersection density (an indicator of navigational complexity and destination accessibility), percentage of commercial land and non-commercial land use mix (indicators of destination diversity and access to services) were found. These environmental attributes were directly or indirectly related to engagement in transportation walking – a behaviour indicative of participation in activities in the local community - which, in turn, showed positive direct effects on cognitive function. Collectively, these findings suggest that dense, destination-rich, interconnected neighbourhoods may benefit cognitive health by promoting behaviours that increase energy expenditure (walking for transport [65]) and neuroplasticity (participation in social, navigational and intellectual activities [23].

Population density as well as street intersection density were negatively related to gardening, likely because home gardens are more common in low density neighbourhoods. As observed earlier, it is interesting that lower frequency of gardening in those who engaged in this activity was predictive of better processing speed. It may be that individuals who, as a result of living in low-density suburbs, spend a lot of time gardening do this at the expense of activity diversity [64] or activities with greater cognitive benefits, such as socialising [23].

As originally hypothesised, not all indirect effects of population density and related built environment attributes on cognitive function were positive. These negative indirect effects were channelled through frequency of transportation walking, possibly capturing inter-individual differences in exposure to ambient air pollution. Individuals who walk for transport on most days of the week are likely to be more exposed to harmful pollutants (roadside pollution) than those who walk less frequently [66]. As a consequence, the cognitive health of the former may benefit less from participation in transportation walking.

Overall, we expected physical activity and sedentary behaviours to be stronger mediators of neighbourhood environment-cognitive function associations than they were in this study. Notwithstanding the fact that measurement error might have attenuated the associations of physical activity and sedentary behaviours with cognitive function, this could be due to the lack of more detailed information about the nature of the activities performed. Evidence suggests that regular engagement in a variety of activities, including physical (e.g., walking), social (e.g., socialising) and intellectual (e.g., learning) activities across life and/or later in life positively contribute to cognitive function [23]. It could be argued, however, that no type of activity, including physical activity, is purely physical, social or cognitive. For example, leisure-time walking may be a predominantly physical (solitary walking on a treadmill) or a predominantly social and cognitive activity (exploring unfamiliar environments with friends while strolling). Sitting for leisure may include or lack a social and/or intellectual component. Activities are likely to have a more beneficial effect on cognitive function if they include all three aspects - physical, social and intellectual. This remains an important topic for future research in this field.

### Strength, Limitations and Further Studies

This study has several strengths. We utilised data from a national study of Australian adults with good geographical coverage and diversity. Unlike previous studies on this topic, we examined the joint linear and/or curvilinear effects of three distinctive but interrelated sets of neighbourhood characteristics (built environment, natural environment and air pollution) on two cognitive functions (memory and processing speed) and the potential role of various physical activity and sedentary behaviours. We addressed reverse causality arising from neighbourhood self-selection by including self-report measures of reasons for living in a neighbourhood as covariates (where appropriate). We based our analytical models on a careful consideration of plausible causal effects among a large range of factors, that were synthesised in the form of directed acyclic graphs.

This study is not void of limitations. Participants in AusDiab3 were healthier than those at baseline (AusDiab1) and this might have attenuated the associations between the examined variables. Although steps to reduce the likelihood of reverse causality were taken, the cross-sectional nature of this study limits the strength of causal evidence. Physical activity and sedentary behaviours were self-reported, increasing the chance of measurement error especially in participants with memory problems. Information on the habitual settings of physical activity and sedentary behaviours was not available. Participants might have undertaken a substantial proportion of their activities outside their neighbourhood. This would have attenuated the effects of the environment on the behaviours. This study employed coarse measures of access to destinations relevant to cognition-enhancing activities because more detailed measures were not available for all geographical regions covered by AusDiab. This shortcoming has made it difficult to disentangle the positive (access to destinations) and negative effects (pollution) of urban densification on cognitive function and related mediators as detailed in the discussion of findings. To address these limitations, future studies would need to: conduct multiple assessments across a relatively extended period of time (>2 years) to capture changes in cognitive function [67] and environmental attributes; collect objective contextual information on physical activity and sedentary behaviours using global positioning system technologies and map-based interviews [68]; and characterise the neighbourhood environment and other activity spaces using more relevant and detailed information on the type and quality of destinations as well as the surrounding landscape and streetscape [18].

## Conclusion

This study has provided partial support for a proposed ecological models of neighbourhood environmental influences on cognitive function. All neighbourhood environmental attributes examined in this study were directly and/or indirectly linked to cognitive function via other environmental attributes or physical activity. The total effects of population density and percentage of parkland were positive, whilst those of other attributes where nil and sometimes contrary to expectations (e.g., positive effect of PM_2.5_ on memory). These unexpected findings were likely due to generally low levels of ambient air pollution and the use of course measures of access to destinations that support cognition-enhancing activities. Engagement in transportation walking and frequency of vigorous gardening partially mediated the positive effects of the neighbourhood environment on cognitive function, while frequency of transportation walking mediated the negative effects. Overall, this study suggests that, possibly due to relatively low levels of ambient air pollution in Australia, the effects of urban densification and related environmental attributes on cognitive function are mainly positive, and might be in part attributed to these neighbourhood features encouraging participation in a diversity of activities.

This study has demonstrated how, to better understand the impacts of the neighbourhood physical environment on cognitive health, key aspects of the built environment, natural environment and air pollution and their interdependencies can be modelled. Apart from the need for longitudinal studies that can more robustly assess causation, future research would benefit from a more accurate characterisation of the neighbourhood environment, physical activity and sedentary behaviours in relation to cognitive function.

## Supplementary Information

**Additional file 1.**

## Data Availability

Data that support the findings of this study are available on request under a license agreement. Written applications can be made to the AusDiab Steering Committee (Dianna.Magliano@baker.edu.au).

## Abbreviations

ABS: Australian Bureau of Statistics
AusDiab: Australian Diabetes, Obesity and Lifestyle study
CI: confidence intervals
CVLT: California Verbal Learning Test
DAG: directed acyclic graph
ESRI: Environmental Systems Research Institute
GAMM: Generalised Additive Mixed Models
IRSAD: Index of Relative Socioeconomic Advantage and Disadvantage
M: mean
NO_2_: nitrogen dioxide
PM_2.5_: particulate matter < 2.5 μm
RMSE: root mean square error
SA1: Statistical Area 1
SD: standard deviation
SDMT: Symbol–Digit Modalities test
VIF: variance inflation factor
