# Supplementary material for: From urban neighbourhood environments to cognitive health: a cross-sectional analysis of the role of physical activity and sedentary behaviours
Source: BMC Public Health. 2021 Dec 23;21:2320. doi: 10.1186/s12889-021-12375-3 (PMC8705462; doi:10.1186/s12889-021-12375-3)
Supplement: Supplementary file 1 — Additional file 1. [file 12889_2021_12375_MOESM1_ESM.docx]

**Additional file 1**

***From urban neighbourhood environments to cognitive health: a cross-sectional analysis of the role of physical activity and sedentary behaviours***

*Authors: Ester Cerin, Anthony Barnett, Jonathan E. Shaw, Erika Martino, Luke D. Knibbs, Rachel Tham, Amanda J. Wheeler and Kaarin J. Anstey*

**Table of Contents**

**S1. Detailed description of built and natural environmental exposures**

**S2. Detailed description of potential mediators**

**S3. Detailed description of analytical steps**

**Figure S1.** Directed acyclic graph (DAG) depicting the hypothesised relations between neighbourhood attributes, physical activity and sedentary behaviours, and cognitive function.

**Table S1.** Outline of regression analyses

**S4. Supplementary results**

**Table S2.** Relationships between neighbourhood environmental variables

**Figure S2.** Relationships between neighbourhood environmental characteristics

**Figure S3.** Curvilinear relationships of population density with street intersection density (top left panel), percentage of commercial land use (top right panel), non-commercial land use mix (bottom left panel) and percentage parkland (bottom right panel) (in 1km residential buffers)

**Figure S4.** Curvilinear relationships of population density with average annual concentrations of NO_2_ (left panel) and PM_2.5_ (right panel)

**Figure S5.** Curvilinear relationships of street intersection density (left panel) and non-commercial land use mix (right panel) with average annual concentrations of NO_2_

**Table S3.** Relationships between environmental characteristics, physical activity and sedentary behaviours – direct effects

**Figure S6.** Effects of environmental characteristics on physical activity (direct) and sedentary behaviours (direct and indirect via physical activity)

**Table S4.** Relationships between environmental characteristics and physical activity – total effects

**Table S5.** Relationships of environmental characteristics, physical activity and sedentary behaviours with cognitive function measures – direct effects

**S1. Detailed description of built and natural environmental characteristics**

Measures of the neighbourhood built and natural environment were generated using ESRI’s ArcGIS v.10.5 software (ESRI, Redlands). Participants’ residential addresses were geocoded and 1-km untrimmed street-network buffers were created around the geocoded locations following procedures employed in international studies of neighbourhood environmental determinants of health-related behaviours and obesity in adults and older adults [1-3]. A 1-km radius was used to create residential buffers because it corresponds to the distance that adults and older adults without mobility problems can cover in a 10-20 minute walk [1], and the latter is commonly used to define a neighbourhood [4, 5].

Four built environment measures were computed for each participant’s residential buffer. These included population density, street intersection density, percentage of commercial land use and an entropy score denoting the heterogeneity of non-commercial land use. *Population density*, here defined as the number of persons per hectare, was derived using the Australian Bureau of Statistics (ABS) Mesh Block data from the 2011 Census [6]. Mesh Blocks are the smallest geographical areas defined by the ABS for which Census data are available. Population density was selected as an environmental exposure of interest because it is the primary driver of changes in the built environment [7, 8] and is consistently linked to active transport [9]. It may also facilitate social activities and confer associated cognitive benefits [10]. *Street intersection density,* defined as the number of ≥3-way intersections per km^2^, was computed using road network data derived from the PSMA Australia’s 2012 Transport & Topography dataset [11]. As this neighbourhood feature provides easier access to public transport and destinations, it is deemed to facilitate walking [7]. However, it may also result in greater personal exposure to air pollution in trafficked areas and, hence, harm residents’ well-being [12]. The *percentage of* buffer area devoted to *commercial land use* (retail and services) was derived from 2011 ABS data on the main land use for Mesh Blocks [13]. Commercial land uses that support good service provision, such as shops and supermarkets provide destinations to which people can walk to from their home and engage in various activities [8, 9]. A land use entropy score or *land use mix* ranging from 0 to 1 [14] and denoting the heterogeneity of non-commercial five land use categories (i.e., residential, industrial, medical, educational and other land uses from 2011 ABS Mesh Block data)[13] was computed to quantify accessibility of various non-commercial destinations that may promote active transport and engagement in activities [7, 15].

Two natural environment measures were included in this study: *percentage of* residential buffer area covered by *parkland* derived from 2011 ABS Mesh Block data [13] and *percentage of* buffer area cover by waterbodies or *blue spaces* (e.g., lakes, coastlines, rivers and reservoirs) derived from national topographic spatial data for surface water features sourced from Geoscience Australia [16]. Access to parks has been relatively consistently associated with higher levels of physical activity [8, 9], better mental health [17] and, in some studies, better cognitive function [18]. Green spaces also mitigate ambient air pollution [19]. Access to fresh water and navigable waterbodies are of crucial importance to humans [20, 21]. A few studies have also found blue space accessibility to promote physical activity [22] and better mental health [23] in part due to its restorative properties (e.g., stress reduction) [24] and its role in promoting social interactions [25].

**S2. Detailed description of potential mediators (measures of physical activity and sedentary behaviours)**

Several potential behavioural mediators of environment-cognitive function associations were examined, including four measures of physical activity and three measures of sedentary behaviour (two of which were combined into one measure). Previous-week frequencies of engagement in transportation walking, leisure-time walking, vigorous gardening and resistance training were gauged using original or modified items from the Active Australia Survey [26]. Frequency rather than total minutes of physical activities were used because the latter are typically associated with greater measurement error [27, 28] and environmental attributes have often been found to be more strongly related to the former [28, 29]. Sedentary behaviour was assessed using items developed for AusDiab3 validated against objectively measured sitting time [30]. Participants were asked to report average daily sitting time in the previous week (h/day) separately for weekdays and weekend days across five domains (occupational, transport, television viewing, leisure-time computer and other domains). For the purpose of this study, occupational and other domain sitting were not examined as mediators of neighbourhood environment-cognitive function associations because they are likely to be performed outside the neighbourhood. Television viewing and leisure-time computer time were combined into a measure of leisure-time sitting.

**References**

1. Adams MA, Frank LD, Schipperijn J, et al. International variation in neighborhood walkability, transit, and recreation environments using geographic information systems: the IPEN adult study. Int J Health Geogr. 2014;13:43. doi: 10.1186/1476-072X-13-43.
2. Cerin E, Van Dyck D, Zhang CJP, et al. Urban environments and objectively-assessed physical activity and sedentary time in older Belgian and Chinese community dwellers: potential pathways of influence and the moderating role of physical function. Int J Behav Nutr Phys Act. 2020;17(1):73. doi: 10.1186/s12966-020-00979-8.
3. Cochrane T, Yu Y, Davey R, et al. Associations of built environment and proximity of food outlets with weight status: Analysis from 14 cities in 10 countries. Prev Med. 2019;129:105874. doi: 10.1016/j.ypmed.2019.105874.
4. Cerin E, Conway TL, Cain KL, et al. Sharing good NEWS across the world: developing comparable scores across 12 countries for the Neighborhood Environment Walkability Scale (NEWS). BMC Public Health. 2013;13:309. doi: 10.1186/1471-2458-13-309.
5. Gunn LD, King TL, Mavoa S, et al. Identifying destination distances that support walking trips in local neighbourhoods. J Trans Health. 2017;5:133-149. doi: 10.1016/j.jth.2016.08.009
6. ABS. Census of population and housing: mesh block counts, 2011 (cat. no. 2074). Canberra: Australian Bureau of Statistics; 2011.
7. Cerin E, Barnett A, Zhang CJP, et al. How urban densification shapes walking behaviours in older community dwellers: a cross-sectional analysis of potential pathways of influence. Int J Health Geogr. 2020;19(1):14. doi: 10.1186/s12942-020-00210-8.
8. Sallis JF, Cerin E, Kerr J, et al. Built environment, physical activity, and obesity: Findings from the International Physical Activity and Environment Network (IPEN) Adult Study. Annu Rev Public Health. 2020;41:119-139. doi: 10.1146/annurev-publhealth-040218-043657.
9. Cerin E, Nathan A, van Cauwenberg J, et al. The neighbourhood physical environment and active travel in older adults: a systematic review and meta-analysis. Int J Behav Nutr Phys Act. 2017;14(1):15. doi: 10.1186/s12966-017-0471-5.
10. Hand CL, Howrey BT. Associations among neighborhood characteristics, mobility limitation, and social participation in late life. J Gerontol B Psychol Sci Soc Sci. 2019;74(3):546-555. doi: 10.1093/geronb/gbw215.
11. PSMA Australia Ltd. PSMA street network; 2012.
12. Zhang CJP, Barnett A, Johnston JM, et al. Objectively-measured neighbourhood attributes as correlates and moderators of quality of life in older adults with different living arrangements: the ALECS cross-sectional study. Int J Environ Res Public Health. 2019;16(5):876. doi: 10.3390/ijerph16050876.
13. ABS. Australian Statistical Geography Standard (ASGS) Volume 1 - Main Structure and Greater Capital City Statistical Areas (cat no. 1270.0.55.001). Canberra: Australian Bureau of Statistics; 2011.
14. Frank LD, Sallis JF, Saelens BE, et al. development of a walkability index: application to the Neighborhood Quality of Life Study. Br. J. Sports Med. 2010;44:924–33.
15. Besser LM, McDonald NC, Song Y, et al. Neighborhood environment and cognition in older adults: a systematic review. Am J Prev Med 2017;53(2):241-251. doi: 10.1016/j.amepre.2017.02.013
16. Crossman S, Li O. Surface Hydrology Polygons (National). Canberra: Geoscience Australia; 2015.
17. Barton J, Rogerson M. The importance of greenspace for mental health. B J Psych Int. 2017;14(4):79-81. doi:10.1192/s2056474000002051.
18. de Keijzer C, Gascon M, Nieuwenhuijsen MJ, et al. Long-term green space exposure and cognition across the life course: a systematic review. Curr Environ Health Rep. 2016;3(4):468–477. doi: 10.1007/s40572-016-0116-x.
19. Hirabayashi S, Nowak DJ. Comprehensive national database of tree effects on air quality and human health in the United States. Environ Pollut. 2016;215:48-57. doi: 10.1016/j.envpol.2016.04.068.
20. Kummu M, de Moel H, Ward PJ, et al. How close do we live to water? A global analysis of population distance to freshwater bodies. PLoS One. 2011;6(6):e20578. doi:10.1371/journal.pone.0020578
21. Tundi A, Alder J. Coastal Systems. In Rashin Hassan, Robert Scholes, and Neville Ash, eds, Ecosystems and Human Well-Being: Current State and Trends, Volume 1, Washington, DC: Island Press; 2005.
22. Pasanen TP, White MP, Wheeler BW, et al. Neighbourhood blue space, health and wellbeing: The mediating role of different types of physical activity. Environ Int. 2019; 131:105016. doi: 10.1016/j.envint.2019.105016.
23. Volker S, Kistemann T. Developing the urban blue: Comparative health responses to blue and green urban spaces in Germany. Health Place. 2011;35:196-205.
24. Gascon M, Triguero-Mas M, Martínez D, et al. Mental health benefits of long-term exposure to residential green and blue spaces: A systematic review. Int J Environ Res Public Health. 2015;12;4354-4379.
25. Maas J, van Dillen SME, Verheij RA, Groenwegen PP. Social contacts as a possible mechanism behind the relation between green space and health. Health Place. 2009;15:586-595. doi: 10.1016/j.healthplace.2008.09.006
26. Australian Institute of Health and Welfare. The Active Australia Survey: a guide and manual for implementation, analysis and reporting. Edited by Welfare AIoHa. Canberra: Australian Institute of Health and Welfare; 2003.
27. Cerin E, Barnett A, Sit CH, et al. Measuring walking within and outside the neighborhood in Chinese elders: reliability and validity. BMC Public Health. 2011;11:851. doi: 10.1186/1471-2458-11-851.
28. Owen N, Cerin E, Leslie E, et al. Neighborhood walkability and the walking behavior of Australian adults. Am J Prev Med. 2007;33(5):387-95. doi: 10.1016/j.amepre.2007.07.025.
29. Cerin E, Sit CH, Barnett A, et al. Ageing in an ultra-dense metropolis: perceived neighbourhood characteristics and utilitarian walking in Hong Kong elders. Public Health Nutr. 2014;17(1):225-32. doi: 10.1017/S1368980012003862.
30. Clark BK, Lynch BM, Winkler EA, et al. Validity of a multi-context sitting questionnaire across demographically diverse population groups: AusDiab3. Int J Behav Nutr Phys Act. 2015;12:148.

**S3. Detailed description of analytical steps**

Descriptive statistics and percentage of missing values were computed for all variables. Over 17% of cases had missing data on at least one variable and 4.5% on more than three variables. Predictors of missingness (the odds of having incomplete data on any of the examined variables) were determined using generalized linear mixed models with binomial variance and logit link functions and random intercepts at the Statistical Area 1 (SA1) level. The odds of having missing data were higher in older participants (*p*<.001), those of non-English speaking background (*p*<.001), with lower household income (*p*=.022), not working or volunteering (*p*=.008), living in areas with lower socio-economic status (*p*=.030) and with lower scores on the memory test (*p*=.036). Missingness was also more prevalent in people for whom access to services was an important reason for living in their neighbourhood (*p*=.040) and those living in areas with higher population density (*p*<.001), lower street intersection density (*p*=.010) and lower concentrations of PM_2.5_ (*p*<.001). As data were at least missing at random (MAR) rather than missing completely at random (MCAR), ten imputed datasets were created for the regression analyses as recommended by Rubin [1] and van Buuren [2]. Multiple imputations by chained equations were performed following currently recommended model-building and diagnostic procedures [2] and using the package ‘mice’ [3] in R version 4.0.0 [4].

The aim of this study was to quantify the potential impact of neighbourhood environment characteristics on cognitive function and the mediating roles of physical activity and sedentary behaviours (i.e. to quantify the total, direct and indirect effects of environmental characteristics on cognitive function). Generalised additive mixed models (GAMMs; package ‘mgcv’ version 1.8.22 [5] in R) with random intercepts at the SA1 level were used for this purpose to account for curvilinear relationships of unknown form and spatially correlated data [5]. Here, the meaning of ‘effect’ needs to be interpreted in the context of the cross-sectional observational nature of the study with possible unmeasured confounders. Directed acyclic graphs (DAGs) were used to inform the selection of a minimal sufficient set of confounders to be included in the GAMMs estimating exposure-outcomes, exposure-mediators and mediators-outcomes relationships (Figure S1). The DAGs were based on the hypothesised causal effects among the variables according to previous studies (see Introduction and Methods sections in the paper and explanation below) and the authors’ expert opinion. Potential multicollinearity was assessed by computing the Variance Inflation Factor (VIF) for each variable included in the GAMMs. All VIFs were smaller than 2.58, indicating no collinearity issues [6]. Analyses were conducted in several steps described below.


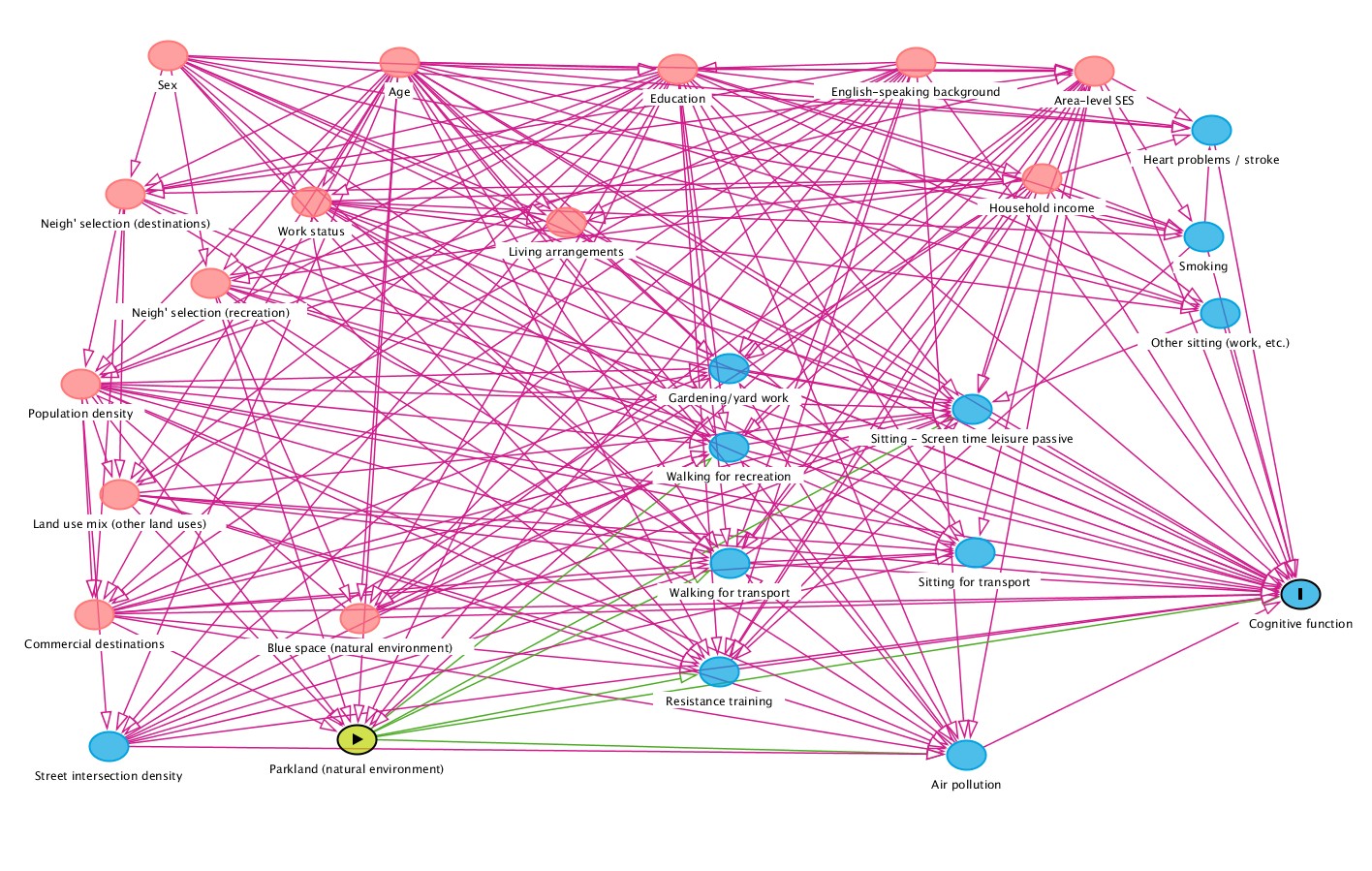


**Figure S1.** Directed acyclic graph (DAG) depicting the hypothesised relations between neighbourhood attributes, physical activity and sedentary behaviours, and cognitive function. Through the DAG, we identified which covariates to include in the statistical analyses to sufficiently control for potential confounders. This particular DAG was used to inform the model of the total effect of percentage of parkland in the neighbourhood on cognitive functions. Variables with red circles denote the set of potential confounders. A minimal sufficient set of confounders (included in the regression models) is a subset of this set of variables.

*Total effects of neighbourhood environmental characteristics on cognitive function*

The total effects of neighbourhood characteristics on each of the two cognitive function outcomes (memory and processing speed) were first estimated. DAGs were used to select a minimal sufficient set of confounders for each of the eight environmental exposures. These are presented in Table S1 (Step 1Ta to 1Th). GAMMs with Gaussian variance and identity link functions were used to model scores on the memory (CVLT) and processing speed (SDMT) tests. Curvilinear associations were estimated using smooth terms modelled with thin plate splines [5]. If the data did not provide sufficient evidence of a curvilinear association, smooth terms were replaced by linear terms. Model selection (linear vs. curvilinear effect) was based on Akaike Information Criterion (AIC) values, where a lower AIC was indicative of a better-fitting model. A median ≥5-unit difference in AIC across all imputed datasets was used as the criterion for linear vs. curvilinear effect selection [7, 8].

We hypothesised that neighbourhood population density, percentage of commercial land use and non-commercial land use mix would be positively related to all measure of cognitive function as they provide opportunities for physical, social and cognitive activities to residents [9-11]. This hypothesis was based on air pollution levels being typically low in Australia [11] and the assumption that increases in air pollution associated with densification would not be sufficiently high to offset the positive effects of environmental complexity and access to destinations. The total effects of percentage of parkland and blue space in the neighbourhood on cognitive function were expected to be positive or, as observed in recent studies, curvilinear [9, 10, 12], and those of air pollution negative [13, 14]. As street intersection density may promote active transport (a potentially beneficial factor for cognitive health) [9, 15, 16] but also increase direct exposure to traffic-related air pollution (a harmful influence) [17], no significant total effects of this environmental attribute on cognitive function were expected.

**Table S1. Outline of regression analyses**

| *Estimation of total effects of neighbourhood environmental attributes on three measures of cognitive function* | | | |
| --- | --- | --- | --- |
| Step | Exposure(s) / effect(s) | Confounders and covariates | Regression models |
| 1Ta* | Population density (persons/hectare) | Age, sex, English-speaking background, living arrangements, educational attainment, area-level IRSAD, residential self-selection related to access to destinations | Two separate sets of GAMMs (one GAMM with a linear and another with a smooth term for the environmental attribute), one set for each cognitive outcome. GAMMs with Gaussian variance and identity link functions. |
| 1Tb* | Street intersection density (intersections/km^2^) | Age, sex, English-speaking background, educational attainment, population density, area-level IRSAD | As above |
| 1Tc* | Percentage of commercial land use (% area in residential buffer) | Age, sex, English-speaking background, educational attainment, population density, area-level IRSAD, residential self-selection related to access to destinations | As above |
| 1Td* | Land use mix (entropy score of 5 non-commercial land uses) | Age, sex, English-speaking background, educational attainment, population density, area-level IRSAD, residential self-selection related to access to destinations | As above |
| 1Te* | Percentage of parkland (% of area in residential buffer) | Age, sex, English-speaking background, educational attainment, population density, percentage of commercial land use, land use mix (5 non-commercial land uses), area-level IRSAD, residential self-selection related to recreational facilities | As above |
| 1Tf* | Percentage of blue space (% of area in residential buffer) | Age, sex, English-speaking background, educational attainment, area-level IRSAD, residential self-selection related to recreational facilities, household income | As above |
| 1Tg* | Annual average NO_2_ exposure (ppb) | Age, sex, English-speaking background, educational attainment, area-level IRSAD, population density, street intersection density, percentage of commercial land use, land use mix (5 non-commercial land uses), percentage of parkland, residential self-selection related to recreational facilities | As above |
| 1Th* | Annual average PM_2.5_ exposure (μg/m^3^) | Age, sex, English-speaking background, educational attainment, area-level IRSAD, population density, street intersection density, percentage of commercial land use, land use mix (5 non-commercial land uses), percentage of parkland, residential self-selection related to recreational facilities | As above |
| *Estimation of direct and mediated effects of neighbourhood environmental attributes on two measures of cognitive function* | | | |
| 1Da | Direct effect of population density [exposure 1] on street intersection density, percentage of commercial land and land use mix (5 land uses) [exposures 2] | Age, educational attainment, English-speaking background; residential self-selection related to access to destinations for percentage of commercial land use and land use mix | Three separate GAMMs, one for each environmental attribute (i.e., exposures 3). GAMMs with Gamma variance and logarithmic link functions. |
| 1Db | Direct effect of population density [exposure 1], percentage of commercial land and land use mix (5 non-commercial land uses) [exposures 2] on percentage of parkland [exposure 3] | Age, educational attainment, English-speaking background, residential self-selection related to recreational facilities | A single GAMM with Gamma variance and logarithmic link functions. |
| 1Dc | Direct effect of exposures 1-3 on two measures of air pollution [exposures 4] | Age, educational attainment, English-speaking background, residential self-selection related to recreational facilities | Two separate GAMMs, one for each air pollution measure (i.e., exposures 4). GAMMs with Gaussian variance and identity link function for PM_2.5_ and Gamma variance and logarithmic link function for NO_2._ |
| 2Da | Direct effects of exposures 1-4 and environmental mediator 1 on transportation walking [lifestyle behaviour 1a] | Age, educational attainment, sex, English-speaking background, household income, work/volunteer status, area-level IRSAD, residential self-selection related to access to destinations | Two separate GAMMs, one for a binary measure (engagement vs. not engagement) and a count measure (non-zero weekly frequency) of physical activity. This was done because there was a higher percentage of zero values for this physical activity measure than expected by a negative binomial distribution. GAMMs with binomial variance and logit link functions were used for the binary measure. GAMMs with negative binomial variance and logarithmic link functions were used for the count measure. |
| 2Db | Direct effects of exposures 1-4 (except for non-commercial land use mix) on leisure-time walking [lifestyle behaviour 1b] | Age, educational attainment, sex, household income, work/volunteer status, area-level IRSAD, residential self-selection related to recreational facilities | As above |
| 2Dc | Direct effects of exposures 1, 2 and 4 (except for land use mix and PM_2.5_) on vigorous gardening [lifestyle behaviour 1c] | Age, educational attainment, English-speaking background, household income, living arrangements, work/volunteer status, area-level IRSAD | As above |
| 2Dd | Direct effects of exposures 1, 2 and 3 (except for street intersection density) on resistance training [lifestyle behaviour 1d] | Age, educational attainment, sex, English-speaking background, household income, work/volunteer status, residential self-selection related to recreational facilities, area-level IRSAD | As above |
| 3Da | Direct effects of exposures 1 and 2, and transportation walking [lifestyle behaviour 1a] on sitting for transport [lifestyle behaviour 2a] | Age, educational attainment, work/volunteer status, residential self-selection related to access to destinations, area-level IRSAD | A single GAMM with Gamma variance and logarithmic link function. |
| 3Db | Direct effects of exposures 1-3 and lifestyle behaviours 1a-1d on sitting for leisure [lifestyle behaviour 2b] | Age, sex, educational attainment, household income, work/volunteer status, residential self-selection related to recreational facilities, area-level IRSAD | A single GAMM with Gamma variance and logarithmic link function. |
|  |  |  |  |
| 4D | Direct effects of exposures 1-4 and lifestyle behaviours 1-2 on two measures of cognitive function (memory and processing speed) [outcomes] | Age, sex, educational attainment, English-speaking background, household income, living arrangements, work/volunteer status, smoking status, history of heart problems / stroke, sitting for other purposes, Neigh' selection (destinations), residential self-selection related to recreational facilities, residential self-selection related to access to destinations, area-level IRSAD | Two separate sets of GAMMs (one GAMM with a linear and another with a smooth term for the environmental attribute), one set for each cognitive outcome. GAMMs with Gaussian variance and identity link functions for CVLT and SDMT. |

*Note.* IRSAD, Index of Relative Social Advantage and Disadvantage; GAMM, generalised additive mixed model

*Mediated and direct effects of neighbourhood environmental characteristics on cognitive function*

Mediation was examined using the joint-significance test [18, 19] according to which data support mediation if the associations (regression coefficients) between an exposure and its mediator(s) and the exposure-adjusted associations between the mediator(s) and the outcome are both statistically significant (*p*<.05). This was done in several steps. First, direct effects of specific environmental attributes on other environmental attributes were estimated (Table S1). This entailed regressing street intersection density, percentage of commercial land and land use mix (5 non-commercial land uses) onto population density (step 1Da in Table S1) for the reasons explained in the Introduction and Environmental exposures sections of the paper. Environmental characteristics potentially influencing the percentage of parkland in residential buffers were examined in step 1Db and those of air pollutants were assessed in step 1Dc. Increases in population density above a certain threshold, and the resulting expansion of residential, commercial, industrial and similar land uses, were hypothesised to lead to a reduction in parkland [18]. Activities resulting from higher levels of population density, street intersection density, commercial land and similar land uses were hypothesised to yield an increase in air pollution [11], while the proportion of parkland was hypothesised to mitigate air pollution levels [20].

In step 2 of the mediation analyses (steps 2Da-2Dd in Table S1), we estimated the direct effects of environmental attributes on measures of physical activity (transportation walking, leisure-time walking, vigorous gardening and resistance training). All examined environmental attributes were considered factors with the potential to influence (positively or negatively) transportation walking [15, 21, 22], and all attributes except for non-commercial land use mix were deemed to potentially influence leisure-time walking [21, 23, 24]. As vigorous gardening typically relies on having a garden at home, we hypothesised that population density and commercial land use would be negatively associated with this activity because high-density areas are typified by apartment blocks rather than detached homes with gardens [25]. Also, we hypothesised that NO_2_ levels and high street intersection density would deter participation in gardening due to these attributes being associated with vehicular traffic [26]. Resistance training may be undertaken in recreational centres, parks or at home. Hence, we hypothesised that better access to parks (percentage of parkland), commercial, educational or medical destinations (non-commercial land use mix and commercial land use) might be positively associated with resistance training. Also, as more densely populated areas may have a higher density of gyms and recreational centres, population density was also deemed to be associated with resistance training.

In step 3 of the mediation analyses (steps 3Da and 3Db in Table S1), we quantified the direct effects of environmental attributes and physical activities on sedentary behaviour. We hypothesised that participants engaging in transportation walking would have lower levels of sitting for transport [27, 28] and that population density, street intersection density, commercial land use and non-commercial land use mix would be negatively related to sitting for transport [27]. Sitting for leisure was expected to be influenced by (negatively related to) all four types of physical activity [28] as well as environmental attributes promoting engagement in activities outside the home (e.g., population density, blue space, parkland and commercial land use) [27]. Step 4 of the mediation analyses estimated the direct effects of environmental factors and physical activity and sedentary behaviours on the two measures of cognitive function (step 4D in Table S1).

**References**

1. Rubin DB. Multiple imputation for non-response in surveys. Wiley J & Sons, New York, 1987.
2. Van Buuren S. Flexible imputation of missing data, 2^nd^ ed. Chapman & Hall, Boca Raton, Florida, 2018.
3. van Buuren S, Groothuis-Oudshoorn K. mice: Multivariate Imputation by Chained Equations in R. J Stat Softw. 2011;45(3):1-67.
4. R Core Team. R: A language and environment for statistical computing. R Foundation for Statistical Computing, Vienna, Austria. 2020. <https://www.R-project.org/>.
5. Wood SN. Generalized additive models: an introduction with R, 2^nd^ ed. Chapman & Hall/CRC, Boca Raton, Florida, 2017.
6. Sheather S. A modern approach to regression with R. Springer, New York, 2009.
7. Burnham KP, Anderson DR. Model selection and multimodel inference: A practical information-theoretic approach, 2^nd^ ed. Springer Verlag, New York 2002.
8. Cerin E, Conway TL, Adams MA, et al. Objectively-assessed neighbourhood destination accessibility and physical activity in adults from 10 countries: An analysis of moderators and perceptions as mediators. Soc Sci Med. 2018;211:282-293. doi: 10.1016/j.socscimed.2018.06.034
9. Besser LM, McDonald NC, Song Y, et al. Neighborhood environment and cognition in older adults: A systematic review. Am J Prev Med. 2017;53(2):241-251. doi: 10.1016/j.amepre.2017.02.013
10. Cerin E. Building the evidence for an ecological model of cognitive health. Health Place. 2019;60:102206. doi: 10.1016/j.healthplace.2019.102206
11. Cerin E, Barnett A, Chaix B, et al. International Mind, Activities and Urban Places (iMAP) study: methods of a cohort study on environmental and lifestyle influences on brain and cognitive health. BMJ Open. 2020;10(3):e036607. doi: 10.1136/bmjopen-2019-036607
12. Wu YT, Prina AM, Jones A, et al. The built environment and cognitive disorders: results from the Cognitive Function and Ageing Study II. Am J Prev Med. 2017;53(1):25-32. doi: 10.1016/j.amepre.2016.11.020
13. Peters R, Ee N, Peters J, et al. Air pollution and dementia: A systematic review. J Alzheimers Dis. 2019;70(s1):S145-S163. doi: 10.3233/JAD-180631
14. Power MC, Adar SD, Yanosky JD, et al. Exposure to air pollution as a potential contributor to cognitive function, cognitive decline, brain imaging, and dementia: A systematic review of epidemiologic research. Neurotoxicology. 2016;56:235-253. doi: 10.1016/j.neuro.2016.06.004
15. Cerin E, Nathan A, van Cauwenberg J, et al. The neighbourhood physical environment and active travel in older adults: a systematic review and meta-analysis. Int J Behav Nutr Phys Act. 2017;14(1):15. doi: 10.1186/s12966-017-0471-5
16. Livingston G, Huntley J, Sommerlad A, et al. Dementia prevention, intervention, and care: 2020 report of the Lancet Commission. Lancet. 2020;396(10248):413-446. doi: 10.1016/S0140-6736(20)30367-6
17. Zhang CJP, Barnett A, Johnston JM, et al. Objectively-measured neighbourhood attributes as correlates and moderators of quality of life in older adults with different living arrangements: The ALECS cross-sectional study. Int J Environ Res Public Health. 2019;16(5):876. doi: 10.3390/ijerph16050876
18. Cerin E, Barnett A, Zhang CJP, et al. How urban densification shapes walking behaviours in older community dwellers: a cross-sectional analysis of potential pathways of influence. Int J Health Geogr. 2020;19(1):14. doi: 10.1186/s12942-020-00210-8
19. MacKinnon DP, Luecken LJ. How and for whom? Mediation and moderation in health psychology. Health Psychol. 2008;27(2S):S99-S100. doi: 10.1037/0278-6133.27.2(Suppl.)
20. Hirabayashi S, Nowak DJ. Comprehensive national database of tree effects on air quality and human health in the United States. Environ Pollut. 2016;215:48-57. doi: 10.1016/j.envpol.2016.04.068
21. Cerin E, Nathan A, Van Cauwenberg, et al. Neighbourhood built environment and older adults’ physical activity. In: Urban Environments for Healthy Ageing: A Global Perspective. Lane AP (Ed.). Routledge, Oxon, UK, 2019.
22. Sallis JF, Cerin E, Kerr J, et al. Built environment, physical activity, and obesity: Findings from the International Physical Activity and Environment Network (IPEN) Adult study. Annu Rev Public Health. 2020;41:119-139. doi: 10.1146/annurev-publhealth-040218-043657
23. Pasanen TP, White MP, Wheeler BW, et al. Neighbourhood blue space, health and wellbeing: The mediating role of different types of physical activity. Environ Int. 2019; 131:105016. doi: 10.1016/j.envint.2019.105016
24. Van Cauwenberg J, Nathan A, Barnett A, et al. Relationships between neighbourhood physical environmental attributes and older adults' leisure-time physical activity: A systematic review and meta-analysis. Sports Med. 2018;48(7):1635-1660. doi: 10.1007/s40279-018-0917-1
25. Saelens BE, Sallis JF, Black JB, Chen D. Neighborhood-based differences in physical activity: an environment scale evaluation. Am J Public Health. 2003;93(9):1552-8. doi: 10.2105/ajph.93.9.1552
26. WHO Regional Office for Europe. Review of evidence on health aspects of air pollution – REVIHAAP Project: Technical Report [Internet]. Copenhagen: WHO Regional Office for Europe; 2013. C, Proximity to roads, NO2, other air pollutants and their mixtures. Available from: <https://www.ncbi.nlm.nih.gov/books/NBK361807/>
27. Barnett A, Cerin E, Ching CS, et al. Neighbourhood environment, sitting time and motorised transport in older adults: a cross-sectional study in Hong Kong. BMJ Open. 2015;5(4):e007557. doi: 10.1136/bmjopen-2014-007557
28. Cerin E, Van Dyck D, Zhang CJP, et al. Urban environments and objectively-assessed physical activity and sedentary time in older Belgian and Chinese community dwellers: potential pathways of influence and the moderating role of physical function. Int J Behav Nutr Phys Act. 2020;17(1):73. doi: 10.1186/s12966-020-00979-8

**S4. Supplementary results**

Table S2 reports the results of the GAMMs estimating the relationships between neighbourhood environmental characteristics, while Fig. S2 shows the hypothesised causal links between these characteristics and the direction of the significant associations found in this study. Fig. S3 to S5 depict the curvilinear relationships mentioned in Table S2.

**Table S2. Relationships between neighbourhood environmental variables**

| **Models** | **Direct effect of …** | **… on (response variable)** | **Statistic** | **Statistic values** | ***p*-value** |
| --- | --- | --- | --- | --- | --- |
| 1Da.1 | Population density (person/ha) | ***Street intersection density*** (intersections/km^2^) | *F*-ratio (df1, df2) | 341.82 (8.72, 4131.28)  see Figure S3 (top left panel) | **<.001** |
| 1Da.2 |  | ***Percentage of commercial land*** | *F*-ratio (df1, df2) | 24.71 (6.37, 4132.63)  see Figure S3 (top right panel) | **<.001** |
| 1Da.3 |  | ***Non-commercial land use mix*** | *F*-ratio (df1, df2) | 10.53 (4.85, 4134.15)  see Figure S3 (bottom left panel) | **<.001** |
| 1Db | Population density (person/ha) | ***Percentage of parkland*** | *F*-ratio (df1, df2) | 13.88 (2.98, 4134.02)  see Figure S3 (bottom right panel) | **<.001** |
|  | Percentage of commercial land |  | e*^b^* (95% CI) | 0.997 (0.992, 1.003) | .306 |
|  | Non-commercial land use mix (entropy score) |  | e*^b^* (95% CI) | 0.691 (0.538, 0.888) | .**004** |
| 1Dc.1 | Population density (person/ha) | ***NO_2_*** (ppb) | *F*-ratio (df1, df2) | 72.83 (7.05, 4124.05)  see Figure S4 (top right panel) | **<.001** |
|  | Street intersection density (intersections/km^2^) |  | *F*-ratio (df1, df2) | 11.45 (1.98, 4124.05)  see Figure S5 (left panel) | **<.001** |
|  | Non-commercial land use mix (entropy score) |  | *F*-ratio (df1, df2) | 44.76 (2.93, 4124.05)  see Figure S5 (right panel) | **<.001** |
|  | Percentage of commercial land |  | e*^b^* (95% CI) | 1.006 (1.005, 1.007) | **<.001** |
|  | Percentage of parkland |  | e*^b^* (95% CI) | 1.0010 (1.0005, 1.0015) | **<.001** |
| 1Dc.2 | Population density (person/ha) | ***PM_2.5_*** (μg/m^3^) | *F*-ratio (df1, df2) | 9.94 (6.88, 4127.12)  see Figure S4 (bottom left panel) | **<.001** |
|  | Street intersection density (intersections/km^2^) |  | *b* (95% CI) | -0.0001 (-0.0006, 0.0004) | .718 |
|  | Non-commercial land use mix (entropy score) |  | *b* (95% CI) | 0.093 (0.030, 0.153) | **.004** |
|  | Percentage of commercial land |  | *b* (95% CI) | 0.004 (0.003, 0.006) | **<.001** |
|  | Percentage of parkland |  | *b* (95% CI) | 0.002 (0.001, 0.002) | **<.001** |

*Notes.* F-ratio, F-ratio for smooth term defining a curvilinear relationship; df = degrees of freedom; *b*, regression coefficient; CI, confidence interval; e*^b^*, exponentiated regression coefficient (from GAMMs with Gamma variance and logarithmic link function).

**Figure S2. Relationships between neighbourhood environmental characteristics**

*
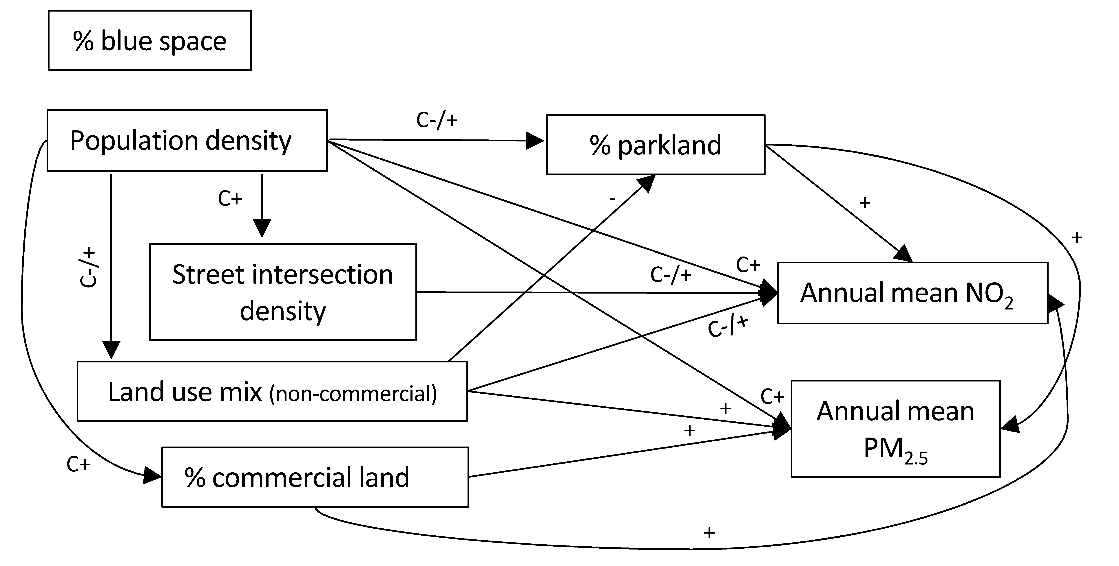
*

*Notes.* Arrows linking variables indicate significant associations. +, -, C, +/- = positive, negative, curvilinear, non-monotonic association. For linear associations, regression coefficients value and their 95% confidence intervals are presented in Table S2. Table S2 provides the F-ratio of the smooth terms for significant curvilinear associations. The curvilinear associations are depicted in Fig. S3 to S5.

**Figure S3. Curvilinear relationships of population density with street intersection density (top left panel), percentage of commercial land use (top right panel), non-commercial land use mix (bottom left panel) and percentage parkland (bottom right panel) (in 1km residential buffers)**


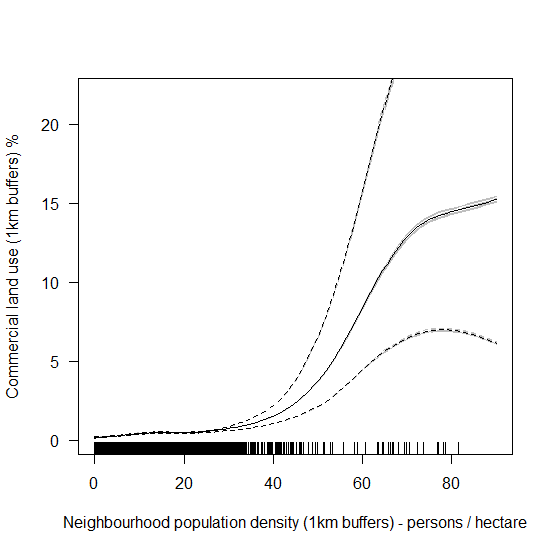

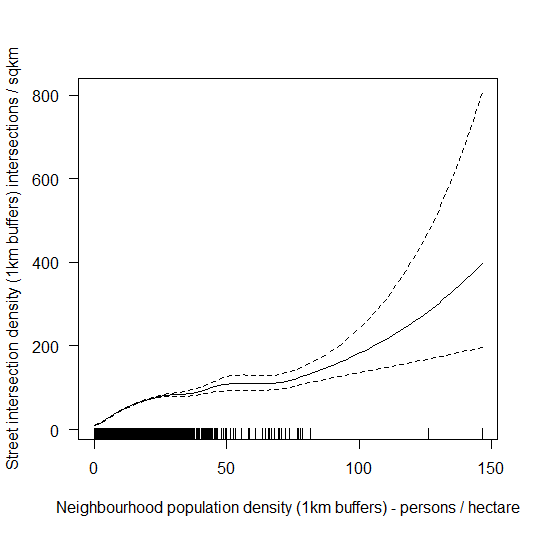


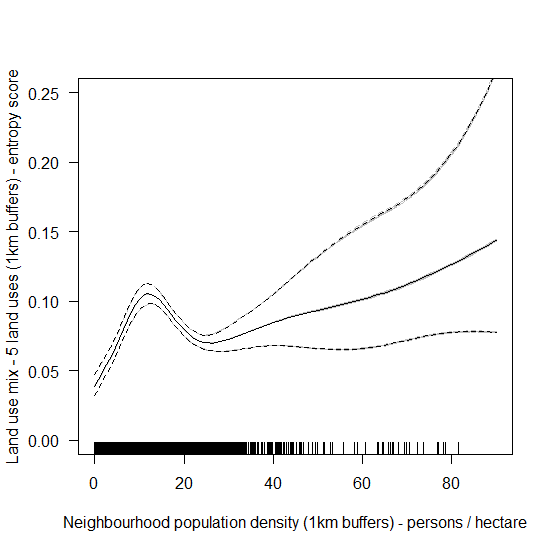

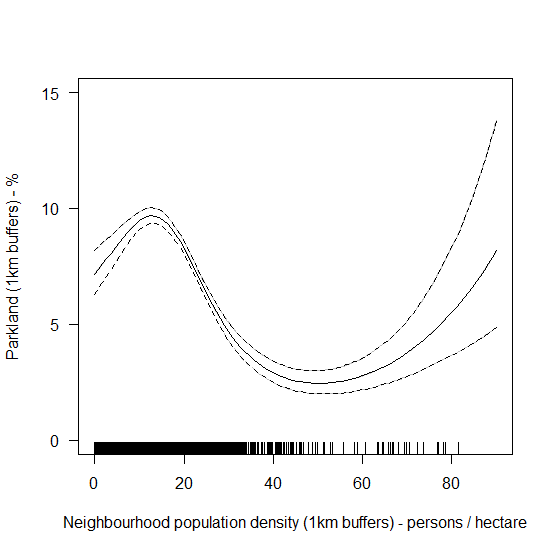


**Figure S4. Curvilinear relationships of population density with average annual concentrations of NO_2_ (left panel) and PM_2.5_ (right panel)**


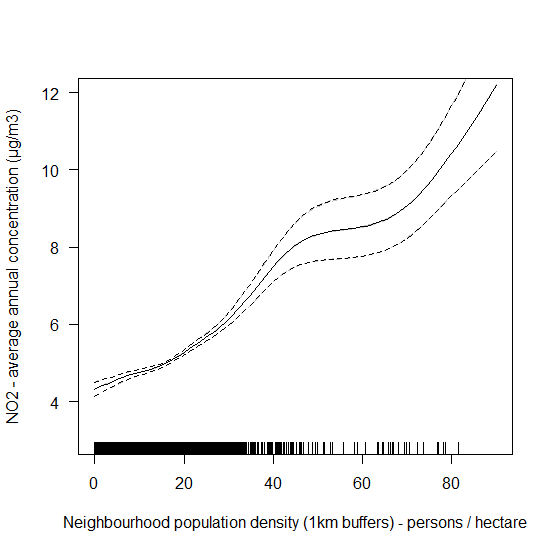

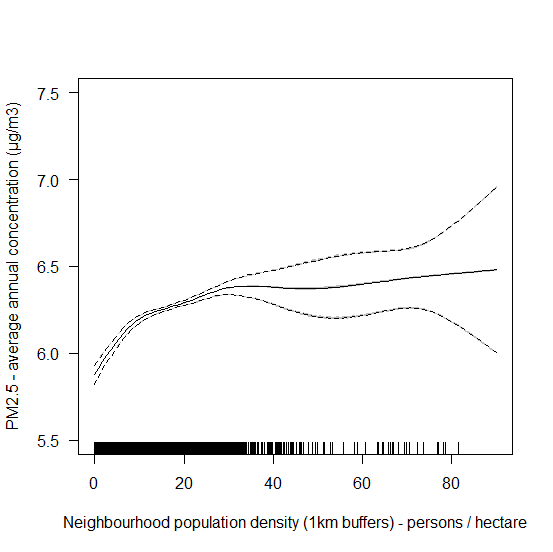


**Figure S5. Curvilinear relationships of street intersection density (left panel) and non-commercial land use mix (right panel) with average annual concentrations of NO_2_**


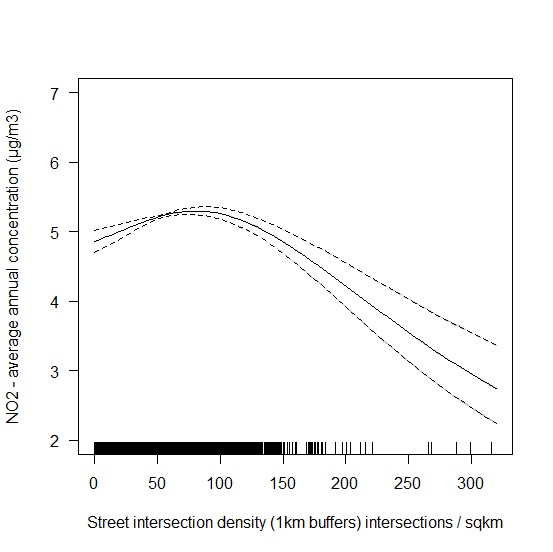

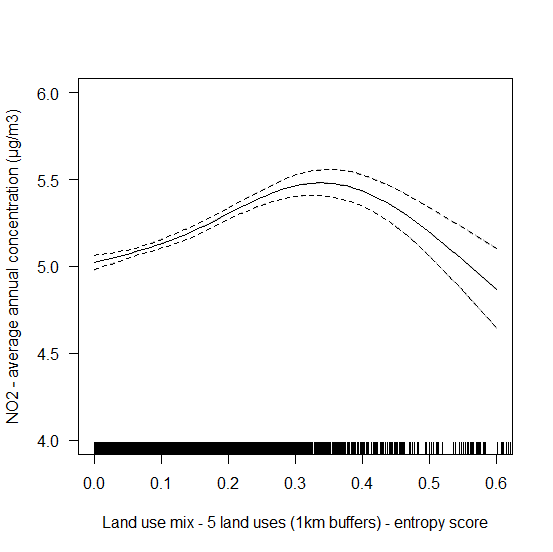


Table S3 shows the results of the GAMMs estimating the direct effects of neighbourhood environmental characteristics on physical activity, and of neighbourhood environmental characteristics and physical activity on sedentary behaviours. Here, the results are presented in the original units of the environmental variables, while the results presented in the main manuscript have been rescaled (e.g., a unit of 10 persons/ha rather than 1 person/ha for population density) to avoid reporting small values for the regression coefficients.

**Table S3. Relationships between environmental characteristics, physical activity and sedentary behaviours – direct effects**

| **Models** | **Direct effect of …** | **… on (response variable)** | **Statistic** | **Statistic values** (original scale) | ***p*-value** |
| --- | --- | --- | --- | --- | --- |
| 2Da.1 | Population density (person/ha) | ***Engagement in transportation walking*** (y/n) | *OR* (95% CI) | 1.007 (0.995, 1.020) | .229 |
|  | Street intersection density (intersection/km^2^) |  | *OR* (95% CI) | 1.004 (1.001, 1.008) | **.004** |
|  | Non-commercial land use mix (entropy score) |  | *OR* (95% CI) | 0.842 (0.436, 1.630) | .611 |
|  | Percentage of commercial land |  | *OR* (95% CI) | 1.018 (1.006, 1.031) | **.005** |
|  | Percentage of parkland |  | *OR* (95% CI) | 0.999 (0.993, 1.006) | .851 |
|  | Percentage of blue space |  | *OR* (95% CI) | 0.949 (0.896, 1.004) | .071 |
|  | NO_2_ (ppb) |  | *OR* (95% CI) | 1.082 (1.028, 1.138) | **.002** |
|  | PM_2.5_ (μg/m^3^) |  | *OR* (95% CI) | 1.110 (1.053, 1.171) | **<.001** |
| 2Da.2 | Population density (person/ha) | ***Frequency of transportation walking**** (times/week) | e*^b^* (95% CI) | 0.999 (0.994, 1.004) | .786 |
|  | Street intersection density (intersection/km^2^) |  | e*^b^* (95% CI) | 1.001 (0.999, 1.002) | .362 |
|  | Non-commercial land use mix (entropy score) |  | e*^b^* (95% CI) | 1.334 (1.002, 1.795) | **.046** |
|  | Percentage of commercial land |  | e*^b^* (95% CI) | 1.006 (1.001, 1.011) | **.022** |
|  | Percentage of parkland |  | e*^b^* (95% CI) | 1.002 (0.999, 1.005) | .175 |
|  | Percentage of blue space |  | e*^b^* (95% CI) | 1.012 (0.977, 1.047) | .518 |
|  | NO_2_ (ppb) |  | e*^b^* (95% CI) | 1.004 (0.983, 1.026) | .707 |
|  | PM_2.5_ (μg/m^3^) |  | e*^b^* (95% CI) | 1.014 (0.989, 1.039) | .278 |
| 2Db.1 | Population density (person/ha) | ***Engagement in leisure-time walking*** (y/n) | *OR* (95% CI) | 0.988 (0.977, 0.999) | **.029** |
|  | Street intersection density (intersection/km^2^) |  | *OR* (95% CI) | 1.005 (1.002, 1.008) | **<.001** |
|  | Percentage of commercial land |  | *OR* (95% CI) | 1.006 (0.994, 1.018) | .334 |
|  | Percentage of parkland |  | *OR* (95% CI) | 0.993 (0.987, 0.999) | **.029** |
|  | Percentage of blue space |  | *OR* (95% CI) | 1.019 (0.981, 1.058) | .326 |
|  | NO_2_ (ppb) |  | *OR* (95% CI) | 1.018 (0.971, 1.068) | .462 |
|  | PM_2.5_ (μg/m^3^) |  | *OR* (95% CI) | 1.001 (0.956, 1.049) | .952 |
| 2Db.2 | Population density (person/ha) | ***Frequency of leisure-time walking**** (times/week) | e*^b^* (95% CI) | 0.999 (0.995, 1.002) | .473 |
|  | Street intersection density (intersections/km^2^) |  | e*^b^* (95% CI) | 1.000 (0.999, 1.001) | .437 |
|  | Percentage of commercial land |  | e*^b^* (95% CI) | 1.002 (0.998, 1.006) | .377 |
|  | Percentage of parkland |  | e*^b^* (95% CI) | 1.000 (0.998, 1.002) | .807 |
|  | Percentage of blue space |  | e*^b^* (95% CI) | 0.999 (0.988, 1.010) | .795 |
|  | NO_2_ (ppb) |  | e*^b^* (95% CI) | 0.993 (0.978, 1.009) | .398 |
|  | PM_2.5_ (μg/m^3^) |  | e*^b^* (95% CI) | 1.000 (0.984, 1.016) | .997 |
| 2Dc.1 | Population density (person/ha) | ***Engagement in vigorous gardening*** (y/n) | *OR* (95% CI) | 1.005 (0.994, 1.016) | .363 |
|  | Street intersection density (intersection/km^2^) |  | *OR* (95% CI) | 0.992 (0.990, 0.995) | **<.001** |
|  | Percentage of commercial land |  | *OR* (95% CI) | 0.993 (0.981, 1.005) | .261 |
|  | Percentage of blue space |  | *OR* (95% CI) | 0.975 (0.938, 1.012) | .251 |
|  | NO_2_ (ppb) |  | *OR* (95% CI) | 0.942 (0.900, 0.987) | **.013** |
| 2Dc.2 | Population density (person/ha) | ***Frequency of vigorous gardening**** (times/week) | e*^b^* (95% CI) | 0.998 (0.992, 1.004) | .478 |
|  | Street intersection density (intersections/km^2^) |  | e*^b^* (95% CI) | 0.997 (0.995, 0.999) | **<.001** |
|  | Percentage of commercial land |  | e*^b^* (95% CI) | 1.006 (0.998, 1.014) | .112 |
|  | Percentage of blue space |  | e*^b^* (95% CI) | 0.975 (0.946, 1.005) | .097 |
|  | NO_2_ (ppb) |  | e*^b^* (95% CI) | 0.985 (0.959, 1.012) | .185 |
| 2Dd.1 | Population density (person/ha) | ***Engagement in resistance training*** (y/n) | *OR* (95% CI) | 1.014 (1.006, 1.022) | **<.001** |
|  | Non-commercial land use mix (entropy score) |  | *OR* (95% CI) | 0.892 (0.492, 1.617) | .707 |
|  | Percentage of commercial land |  | *OR* (95% CI) | 1.001 (0.989, 1.013) | .906 |
|  | Percentage of parkland |  | *OR* (95% CI) | 1.008 (1.002, 1.014) | **.012** |
| 2Dd.2 | Population density (person/ha) | ***Frequency of resistance training*** (times/week) | e*^b^* (95% CI) | 1.001 (0.997, 1.005) | .570 |
|  | Non-commercial land use mix (entropy score) |  | e*^b^* (95% CI) | 0.995 (0.722, 1.372) | .976 |
|  | Percentage of commercial land |  | e*^b^* (95% CI) | 0.998 (0.992, 1.004) | .592 |
|  | Percentage of parkland |  | e*^b^* (95% CI) | 1.001 (0.998, 1.005) | .369 |
| 3Da | Population density (person/ha) | ***Sitting for transport*** (h/day) | e*^b^* (95% CI) | 1.001 (0.997, 1.005) | .534 |
|  | Street intersection density (intersection/km^2^) |  | e*^b^* (95% CI) | 0.999 (0.998, 1.001) | .436 |
|  | Non-commercial land use mix (entropy score) |  | e*^b^* (95% CI) | 0.891 (0.684, 1.161) | .392 |
|  | Percentage of commercial land |  | e*^b^* (95% CI) | 0.992 (0.987, 0.998) | **.004** |
|  | Engagement in transportation walking (ref: no) |  | e*^b^* (95% CI) | 0.978 (0.875, 1.092) | .695 |
|  | Frequency of transportation walking (times/week) |  | e*^b^* (95% CI) | 1.008 (0.984, 1.032) | .541 |
| 3Db | Population density (person/h) | ***Leisure-time sitting*** (h/day) | e*^b^* (95% CI) | 1.003 (0.999, 1.005) | .065 |
|  | Street intersection density (intersection/km^2^) |  | e*^b^* (95% CI) | 0.999 (0.998, 0.999) | **.001** |
|  | Non-commercial land use mix (entropy score) |  | e*^b^* (95% CI) | 0.911 (0.767, 1.081) | .286 |
|  | Percentage of commercial land |  | e*^b^* (95% CI) | 1.002 (0.999, 1.006) | .162 |
|  | Percentage of parkland |  | e*^b^* (95% CI) | 0.999 (0.997, 1.000) | .130 |
|  | Percentage of blue space |  | e*^b^* (95% CI) | 0.985 (0.975, 0.995) | **.004** |
|  | Engagement in transportation walking (ref: no) |  | e*^b^* (95% CI) | 0.882 (0.816, 0.953) | **.002** |
|  | Frequency of transportation walking (times/week) |  | e*^b^* (95% CI) | 1.008 (0.992, 1.026) | .330 |
|  | Engagement in leisure-time walking (ref: no) |  | e*^b^* (95% CI) | 0.953 (0.894, 1.015) | .136 |
|  | Frequency of leisure-time walking (times/week) |  | e*^b^* (95% CI) | 1.001 (0.989, 1.014) | .809 |
|  | Engagement in vigorous gardening (ref: no) |  | e*^b^* (95% CI) | 1.022 (0.963, 1.085) | .472 |
|  | Frequency of vigorous gardening (times/week) |  | e*^b^* (95% CI) | 0.971 (0.951, 0.993) | **.009** |
|  | Engagement in resistance training (ref: no) |  | e*^b^* (95% CI) | 0.916 (0.846, 0.992) | .**030** |
|  | Frequency of resistance training (times/week) |  | e*^b^* (95% CI) | 1.013 (0.994, 1.034) | .186 |

*Note.* * in those engaging in the specific physical activity. *OR*, odds ratio; *b*, regression coefficient; CI, confidence interval; e*^b^*, exponentiated regression coefficient (from GAMMs with Gamma variance and logarithmic link function).

**Figure S6. Effects of environmental characteristics on physical activity (direct) and sedentary behaviours (direct and indirect via physical activity)**


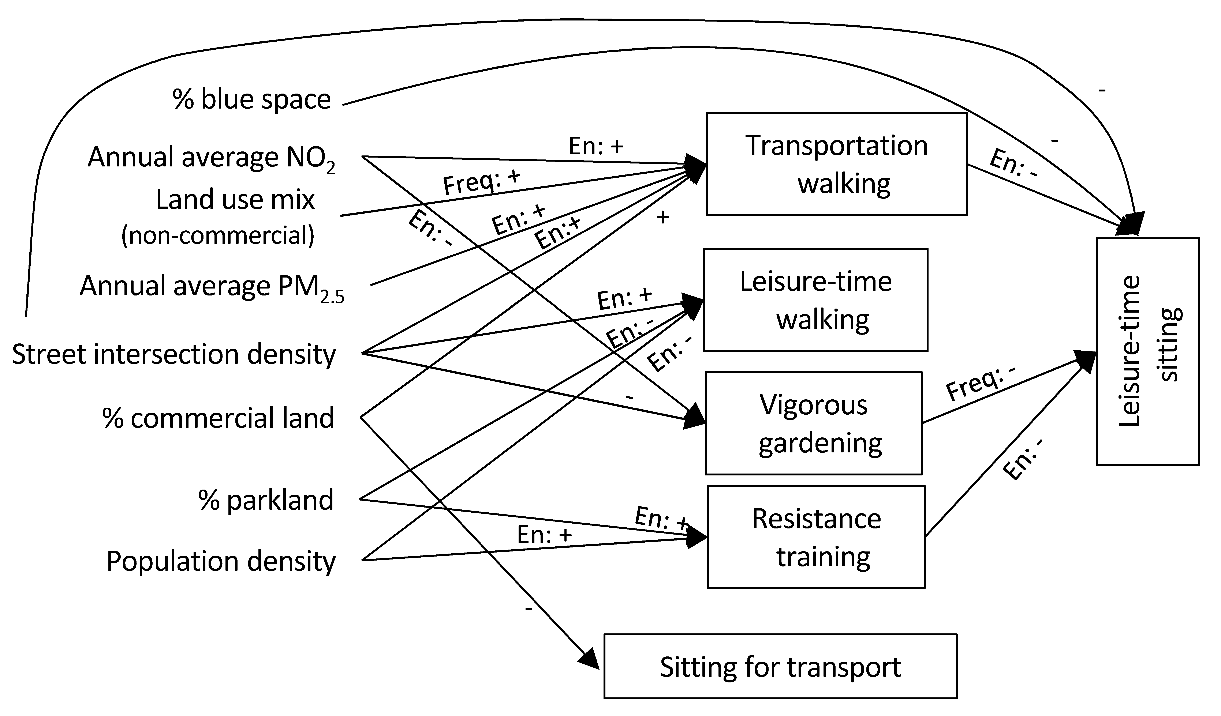


*Notes.* Arrows linking variables indicate significant associations. +, - = positive, negative association; En, Freq = engagement in, frequency in a specific type of physical activity. Regression coefficients value and their 95% confidence intervals are presented in Table S3.

We found that street intersection density was positively, and percentage of parkland and population density were negatively, related to leisure-time walking. NO_2_ and street intersection density were negatively related to engagement in vigorous gardening, while population density and percentage of parkland were positively related to engagement in resistance training. Direct negative effects of percentage of blue space and street intersection density on leisure-time sitting, and of percentage of commercial land on sitting for transport, were observed. All environmental attributes apart from percentage of blue space showed indirect effects on leisure-time sitting. The indirect effects channelled through transportation walking and resistance training were negative (i.e., an increase in a specific environmental attribute was associated with higher levels of physical activity, which, in turn, were associated with less sitting), while those via vigorous gardening were negative (environmental attributes negatively associated with vigorous gardening, which was negatively related to leisure-time sitting).

Table S4 shows the results of the GAMMs estimating the total effects of neighbourhood environmental characteristics on physical activity. Here, the results are presented in the original units of the environmental variables, while the results presented in the main manuscript have been rescaled (e.g., a unit of 10 persons/ha rather than 1 person/ha for population density) to avoid reporting small values for the regression coefficients.

**Table S4. Relationships between environmental characteristics and physical activity – total effects**

| **Models** | **Total effect of …** | **… on (response variable)** | **Statistic** | **Statistic values** (original scale) | ***p*-value** |
| --- | --- | --- | --- | --- | --- |
| 2Ta.1 | Population density (person/ha) | ***Engagement in transportation walking*** (y/n) | *OR* (95% CI) | 1.034 (1.026, 1.043) | **<.001** |
|  | Street intersection density (intersection/km^2^) |  | *OR* (95% CI) | 1.004 (1.001, 1.008) | **.005** |
|  | Non-commercial land use mix (entropy score) |  | *OR* (95% CI) | 1.327 (0.711, 2.479) | .374 |
|  | Percentage of commercial land |  | *OR* (95% CI) | 1.027 (1.014, 1.039) | **<.001** |
|  | Percentage of parkland |  | *OR* (95% CI) | 1.001 (0.995, 1.008) | .672 |
|  | Percentage of blue space |  | *OR* (95% CI) | 0.951 (0.898, 1.006) | .075 |
|  | NO_2_ (ppb) |  | *OR* (95% CI) | 1.103 (1.048, 1.161) | **<.001** |
|  | PM_2.5_ (μg/m^3^) |  | *OR* (95% CI) | 1.114 (1.055, 1.175) | **<.001** |
| 2Ta.2 | Population density (person/ha) | ***Frequency of transportation walking**** (times/week) | e*^b^* (95% CI) | 1.002 (0.999, 1.005) | .301 |
|  | Street intersection density (intersection/km^2^) |  | e*^b^* (95% CI) | 1.001 (0.999, 1.002) | .360 |
|  | Non-commercial land use mix (entropy score) |  | e*^b^* (95% CI) | 1.367 (1.024, 1.825) | **.034** |
|  | Percentage of commercial land |  | e*^b^* (95% CI) | 1.007 (1.002, 1.012) | **.006** |
|  | Percentage of parkland |  | e*^b^* (95% CI) | 1.003 (0.999, 1.006) | .088 |
|  | Percentage of blue space |  | e*^b^* (95% CI) | 1.013 (0.978, 1.049) | .475 |
|  | NO_2_ (ppb) |  | e*^b^* (95% CI) | 1.011 (0.989, 1.033) | .328 |
|  | PM_2.5_ (μg/m^3^) |  | e*^b^* (95% CI) | 1.015 (0.990, 1.041) | .247 |
| 2Tb.1 | Population density (person/ha) | ***Engagement in leisure-time walking*** (y/n) | *OR* (95% CI) | 1.003 (0.996, 1.010) | .404 |
|  | Street intersection density (intersection/km^2^) |  | *OR* (95% CI) | 1.005 (1.002, 1.008) | **<.001** |
|  | Percentage of commercial land |  | *OR* (95% CI) | 1.007 (0.995, 1.019) | .283 |
|  | Percentage of parkland |  | *OR* (95% CI) | 0.994 (0.988, 1.000) | **.039** |
|  | Percentage of blue space |  | *OR* (95% CI) | 1.016 (0.979, 1.055) | .390 |
|  | NO_2_ (ppb) |  | *OR* (95% CI) | 1.015 (0.968, 1.064) | .533 |
|  | PM_2.5_ (μg/m^3^) |  | *OR* (95% CI) | 1.004 (0.959, 1.051) | .869 |
| 2Tb.2 | Population density (person/ha) | ***Frequency of leisure-time walking**** (times/week) | e*^b^* (95% CI) | 0.999 (0.996, 1.001) | .303 |
|  | Street intersection density (intersections/km^2^) |  | e*^b^* (95% CI) | 1.001 (1.000, 1.002) | **.047** |
|  | Percentage of commercial land |  | e*^b^* (95% CI) | 1.002 (0.998, 1.006) | .365 |
|  | Percentage of parkland |  | e*^b^* (95% CI) | 1.000 (0.998, 1.002) | .749 |
|  | Percentage of blue space |  | e*^b^* (95% CI) | 1.000 (0.989, 1.012) | .931 |
|  | NO_2_ (ppb) |  | e*^b^* (95% CI) | 0.992 (0.977, 1.008) | .336 |
|  | PM_2.5_ (μg/m^3^) |  | e*^b^* (95% CI) | 0.999 (0.983, 1.015) | .869 |
| 2Tc.1 | Population density (person/ha) | ***Engagement in vigorous gardening*** (y/n) | *OR* (95% CI) | 0.985 (0.978, 0.993) | **<.001** |
|  | Street intersection density (intersection/km^2^) |  | *OR* (95% CI) | 0.993 (0.990, 0.995) | **<.001** |
|  | Percentage of commercial land |  | *OR* (95% CI) | 0.990 (0.978, 1.002) | .095 |
|  | Percentage of blue space |  | *OR* (95% CI) | 0.972 (0.936, 1.010) | .152 |
|  | NO_2_ (ppb) |  | *OR* (95% CI) | 0.945 (0.902, 0.990) | **.018** |
| 2Tc.2 | Population density (person/ha) | ***Frequency of vigorous gardening**** (times/week) | e*^b^* (95% CI) | 0.995 (0.978, 0.993) | **<.001** |
|  | Street intersection density (intersections/km^2^) |  | e*^b^* (95% CI) | 0.998 (0.996, 0.999) | **<.012** |
|  | Percentage of commercial land |  | e*^b^* (95% CI) | 1.006 (0.998, 1.014) | .149 |
|  | Percentage of blue space |  | e*^b^* (95% CI) | 0.960 (0.931, 0.990) | **.009** |
|  | NO_2_ (ppb) |  | e*^b^* (95% CI) | 0.988 (0.960, 1.016) | .401 |
| 2Td.1 | Population density (person/ha) | ***Engagement in resistance training*** (y/n) | *OR* (95% CI) | 1.014 (1.006, 1.022) | **<.001** |
|  | Non-commercial land use mix (entropy score) |  | *OR* (95% CI) | 0.520 (0.285, 0.947) | **<.001** |
|  | Percentage of commercial land |  | *OR* (95% CI) | 1.001 (0.989, 1.013) | .841 |
|  | Percentage of parkland |  | *OR* (95% CI) | 1.011 (1.005, 1.017) | **<.001** |
|  | Percentage of blue space |  | *OR* (95% CI) | 1.020 (0.987, 1.054) | .240 |
| 2Td.2 | Population density (person/ha) | ***Frequency of resistance training*** (times/week) | e*^b^* (95% CI) | 1.000 (0.996, 1.004) | .960 |
|  | Non-commercial land use mix (entropy score) |  | e*^b^* (95% CI) | 1.174 (0.856, 1.611) | .320 |
|  | Percentage of commercial land |  | e*^b^* (95% CI) | 0.998 (0.992, 1.004) | .570 |
|  | Percentage of blue space |  | e*^b^* (95% CI) | 0.996 (0.981, 1.011) | .606 |
|  | Percentage of parkland |  | e*^b^* (95% CI) | 1.001 (0.998, 1.004) | .618 |

*Note.* * in those engaging in the specific physical activity. *OR*, odds ratio; *b*, regression coefficient; CI, confidence interval; e*^b^*, exponentiated regression coefficient (from GAMMs with Gamma variance and logarithmic link function).

Table S5 shows the results of the GAMMs estimating the direct effects of neighbourhood environmental characteristics, physical activity and sedentary behaviours on cognitive functions. Here, the results are presented in the original units of the environmental variables, while the results presented in the main manuscript have been rescaled (e.g., a unit of 10 persons/ha rather than 1 person/ha for population density) to avoid reporting small values for the regression coefficients.

**Table S5. Relationships of environmental characteristics, physical activity and sedentary behaviours with cognitive function measures – direct effects**

| **Models** | **Direct effect of …** | **… on (response variable)** | | **Statistic** | **Statistic values** (original scale) | ***p*-value** |
| --- | --- | --- | --- | --- | --- | --- |
| 4D.1 | Population density (person/ha) | ***Memory*** (CVLT raw score) | *b* (95% CI) | | 0.007 (-0.005, 0.018) | .235 |
|  | Street intersection density (intersection/km^2^) |  | *b* (95% CI) | | 0.001 (-0.002, 0.004) | .626 |
|  | Percentage of commercial land |  | *b* (95% CI) | | -0.005 (-0.018, 0.007) | .393 |
|  | Non-commercial land use mix (entropy score) |  | *b* (95% CI) | | -0.140 (-0.776, 0.495) | .665 |
|  | Percentage of parkland |  | *b* (95% CI) | | 0.008 (0.001, 0.014) | **.016** |
|  | Percentage of blue space |  | *b* (95% CI) | | -0.010 (-0.044, 0.024) | .576 |
|  | NO_2_ (ppb) |  | *b* (95% CI) | | 0.007 (-0.043, 0.057) | .776 |
|  | PM_2.5_ (μg/m^3^) |  | *b* (95% CI) | | 0.085 (0.035, 0.135) | **<.001** |
|  | Engagement in transportation walking (ref: no) |  | *b* (95% CI) | | 0.412 (0.159, 0.664) | **<.001** |
|  | Frequency of transportation walking (times/week) |  | *b* (95% CI) | | -0.058 (-0.113, -0.003) | **.021** |
|  | Engagement in leisure-time walking (ref: no) |  | *b* (95% CI) | | 0.126 (-0.078, 0.331) | .227 |
|  | Frequency of leisure-time walking (times/week) |  | *b* (95% CI) | | -0.034 (-0.073, 0.005) | .088 |
|  | Engagement in vigorous gardening (ref: no) |  | *b* (95% CI) | | 0.068 (-0.127, 0.264) | .493 |
|  | Frequency of vigorous gardening (times/week) |  | *b* (95% CI) | | -0.025 (-0.092, 0.043) | .474 |
|  | Engagement in resistance training (ref: no) |  | *b* (95% CI) | | 0.177 (-0.079, 0.432) | .175 |
|  | Frequency of resistance training (times/week) |  | *b* (95% CI) | | -0.024 (-0.088, 0.039) | .452 |
|  | Sitting for transport (h/day) |  | *b* (95% CI) | | 0.039 (-0.045, 0.123) | .364 |
|  | Leisure-time sitting (h/day) |  | *b* (95% CI) | | -0.023 (-0.077, 0.031) | .408 |
| 4D.2 | Population density (person/ha) | ***Processing speed*** (SDMT raw score) | *b* (95% CI) | | -0.007 (-0.051, 0.038) | .681 |
|  | Street intersection density (intersection/km^2^) |  | *b* (95% CI) | | -0.001 (-0.011, 0.011) | .990 |
|  | Percentage of commercial land |  | *b* (95% CI) | | -0.007 (-0.055, 0.041) | .765 |
|  | Non-commercial land use mix (entropy score) |  | *b* (95% CI) | | 1.108 (-1.209, 3.424) | .347 |
|  | Percentage of parkland |  | *b* (95% CI) | | 0.021 (0.002, 0.040) | **.048** |
|  | Percentage of blue space |  | *b* (95% CI) | | 0.049 (-0.089, 0.187) | .484 |
|  | NO_2_ (ppb) |  | *b* (95% CI) | | 0.218 (0.032, 0.404) | **.022** |
|  | PM_2.5_ (μg/m^3^) |  | *b* (95% CI) | | -0.058 (-0.238, 0.121) | .525 |
|  | Engagement in transportation walking (ref: no) |  | *b* (95% CI) | | 0.944 (0.119, 1.769) | **.011** |
|  | Frequency of transportation walking (times/week) |  | *b* (95% CI) | | -0.066 (-0.296, 0.163) | .571 |
|  | Engagement in leisure-time walking (ref: no) |  | *b* (95% CI) | | 0.311 (-0.559, 1.180) | .484 |
|  | Frequency of leisure-time walking (times/week) |  | *b* (95% CI) | | -0.138 (-0.303, 0.028) | .104 |
|  | Engagement in vigorous gardening (ref: no) |  | *b* (95% CI) | | 0.193 (-0.610, 0.996) | .637 |
|  | Frequency of vigorous gardening (times/week) |  | *b* (95% CI) | | -0.234 (-0.463, -0.011) | **.045** |
|  | Engagement in resistance training (ref: no) |  | *b* (95% CI) | | -0.269 (-1.335, 0.803) | .626 |
|  | Frequency of resistance training (times/week) |  | *b* (95% CI) | | 0.223 (0.036, 0.410) | **.039** |
|  | Sitting for transport (h/day) |  | *b* (95% CI) | | 0.255 (-0.094, 0.604) | .152 |
|  | Leisure-time sitting (h/day) |  | *b* (95% CI) | | 0.098 (-0.119, 0.315) | .378 |

*Note.* * in those engaging in the specific physical activity. *OR*, odds ratio; *b*, regression coefficient; CI, confidence interval; e*^b^*, exponentiated regression coefficient (from GAMMs with Gamma variance and logarithmic link function).
